# Nanoelectrum across the Visible Spectrum: Tailored Ag–Au Alloy Nanoparticles with Glutathione-Enhanced Stability

**DOI:** 10.1021/acs.chemmater.5c02141

**Published:** 2025-12-06

**Authors:** Matthew G. Ellis, Oriol Colomer I. Ferrer, Muhamad Hartono, Olga Niarchou, Ali Zarkesh, Tijmen G. Euser, Ljiljana Fruk

**Affiliations:** † Nano Photonics Centre, Cavendish Laboratory, Department of Physics, 2152 University of Cambridge, JJ Thomson Avenue, Cambridge CB3 0HE, U.K.; ‡ Department of Chemical Engineering and Biotechnology, University of Cambridge, Phillipa Fawcett Drive, Cambridge CB3 0AS, U.K.; § Hitachi Cambridge Laboratory, Hitachi Europe Ltd, J. J. Thomson Avenue, Cambridge CB3 0HE, U.K.

## Abstract

Nanoelectrum (Ag–Au alloy nanoparticles, Ag–Au NPs) combines the unique nanoscale characteristics of silver and gold into a single bimetallic system exhibiting synergistic properties distinct from those of individual metals. Despite their significant potential and enhanced performance in applications such as catalysis and surface-enhanced Raman spectroscopy (SERS), the use of Ag–Au NPs remains limited due to challenges in achieving controlled and reproducible synthesis. Here, we present surfactant-assisted and surfactant-free approaches for the synthesis of both hollow and solid Ag–Au NPs from AgNP seeds across a wide range of Ag to Au ratios and AgNP sizes. We highlight the synergistic role of Cl^–^ ions and reducing agents in controlling the extent of galvanic replacement and minimizing the loss of Ag. In addition, we demonstrate for the first time the use of glutathione (GSH) as a postsynthetic capping agent to preserve structural integrity and ensure long-term colloidal stability of hollow Ag–Au NPs. These findings provide a much needed versatile and practical framework for fine-tuning the size, shape, and composition of Ag–Au NPs, facilitating the rational design of plasmonic bimetallic systems across the visible spectrum.

## Graphical abstract

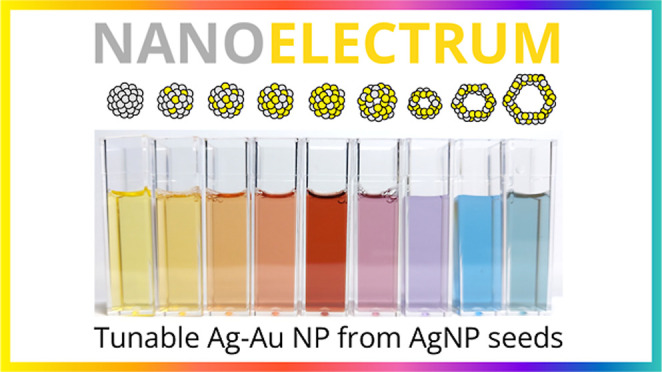

## Introduction

Plasmonic nanoparticles are widely used in applications such as photocatalysis, biosensing, and biomedicine due to their exceptional catalytic and light-harvesting properties.^1–4^ Gold nanoparticles (AuNPs) dominate these fields because of their straightforward synthesis, commercial availability, and favorable balance between their plasmonic properties and long-term chemical stability. In contrast, silver nanoparticles (AgNPs) have superior electromagnetic field enhancement, higher extinction coefficients, and lower cost when compared to AuNPs,^5^ but their practical application is limited by their susceptibility to oxidative dissolution, which significantly compromises their plasmonic properties.^6^

In nature, silver and gold can form an alloy known as electrum, historically recognized as the first known coinage material.^7^ Inspired by this, nanoscale electrum or nanoelectrum (Ag–Au alloy nanoparticles) has attracted significant attention for its ability to deliver properties superior to either metal alone, enabling development of next-generation plasmonic materials.^8–10^

Unlike bimetallic core@shell nanoparticles, fully alloyed Ag–Au nanoelectrum exhibits plasmonic properties distinct from the combination of individual metals.^11^ For example, a characteristic, single localized surface plasmon resonance (LSPR) peak is observed, which can be tuned between the typical wavelengths for AgNPs (∼400 nm) and AuNPs (∼520) by adjusting the Ag/Au ratios.^12^ Such tunability has been utilized for high-performance sensor design and catalysis. Ha Pham et al. reported that Ag_75_Au_25_ NPs allowed for ultrasensitive surface-enhanced Raman spectroscopy (SERS) detection of rhodamine B at concentrations as low as 1 × 10^–11^ M, surpassing pure Ag or Au NPs, while maintaining 80% activity over one year of storage, highlighting the role of Au in enhancing chemical stability without sacrificing sensitivity.^13^ Similarly, Ag_50_Au_50_NPs exhibited superior catalytic activity for the reduction of 4-nitrothiophenol (4-NTP) compared to monometallic nanoparticles, while also allowing for the in situ reaction monitoring using SERS.^14^

However, despite the promise, the adoption of Ag–Au NPs remains limited due to the challenges in achieving precise control over size, shape, and composition, key parameters that determine plasmonic and catalytic properties. Co-reduction of Ag and Au precursors is the most common synthesis approach, but it offers limited tunability. While seed-mediated growth provides greater control for monometallic systems,^15^ adapting this strategy to bimetallic NPs requires additional considerations. For instance, adding Ag to Au seeds typically results in core@shell structures rather than homogeneous alloys,^16^ while simultaneous addition of Ag and Au to Au seeds produces phase-segregated alloyed nanoparticles with an Ag-enriched surface.^17^ Thermal annealing can induce alloying of core@shell nanoparticles but requires high temperatures (∼250 °C), limiting scalability and increasing production cost.^18,19^ Due to higher reduction potential of Au compared to Ag, introduction of Au to Ag NPs triggers a galvanic replacement reaction, in which three Ag atoms are oxidized for every Au atom reduced, generating hollow nanostructures as Ag migrates to the surface to compensate for vacancies through the Kirkendall effect.^20,21^ These hollow nanoparticles exhibit high surface area to volume ratio in addition to LSPR bands extending into near-infrared, advantageous for catalysis and photothermal therapy.^22–24^ This red-shift mainly arises from plasmon hybridization between the inner and outer surfaces, as well as geometric effects introduced by the hollow structure.^25^ Fine control over galvanic replacement can be achieved by adding reducing agents to tune reaction kinetics and limit Ag loss,^26–28^ while capping agents can influence morphology by selectively protecting the Ag surface and directing the galvanic replacement process toward specific crystal facets.^29–31^

Although alloy formation mechanisms are well understood,^32–34^ most reported protocols are optimized for specific ranges of nanoparticle sizes, shapes, and compositions, limiting broader applicability. Moreover, ensuring long-term structural stability, particularly for hollow architectures, remains a challenge. Given that morphology critically governs catalytic and optical properties, there is a need for comprehensive, systematic approaches that enable broad tunability in terms of size, shape, and composition as well as structural robustness. In this work, we address these challenges by presenting a surfactant-free approach using NaCl as a capping agent and 4-(2-hydroxyethyl)-1-piperazineethanesulfonic acid (HEPES) buffer as a reductant. The use of chloride ions played a key role in sufficiently capping Ag seed surfaces to limit the extent of galvanic replacement, and the performance of this method was favorably compared to a surfactant-assisted approach using hexadecyltrimethylammonium chloride (CTAC). We further demonstrate, for the first time, the use of glutathione (GSH) as a postsynthetic protective capping agent to suppress reshaping and ensure long-term stability of hollow Ag–Au NPs. This was found to be of particular importance to surfactant-free Ag–Au NPs, where chloride-capping alone was not sufficient to preserve the stability of larger Ag–Au NPs. Both approaches allow fine modulation of LSPR across the entire visible spectrum (400–700 nm) using AgNP seeds ranging from 16 to 71 nm, offering a practical and scalable platform for designing alloyed plasmonic nanostructures that can be used in the design of next-generation sensing devices and plasmonic catalysts.

## Experimental Section

### Materials and Methods

Sodium citrate tribasic (S4641, Sigma-Aldrich), tannic acid (403040, Sigma-Aldrich), silver nitrate (CHE3244, Scientific Laboratory Supplies), hexadecyltrimethylammonium chloride (411410050, Thermo Scientific), ascorbic acid (A7506, Sigma-Aldrich), gold­(III) chloride trihydrate (G4022, Sigma-Aldrich), 1 M HEPES solution (H0887, Sigma-Aldrich), and sodium chloride (10598630, Fisher) were used. UV–vis characterization of the nanoparticles was carried out with a Shimadzu UV-3600i Plus. Electron microscopy imaging was carried out using a Talos F200X G2 electron microscope (Thermo Scientific) at an operating voltage of 200 kV.

### Synthesis of Ag Nanoparticle Seeds

AgNPs were synthesized according to a previously reported protocol.^35^ Aqueous stock solutions of 100 mM sodium citrate, 2.5 mM tannic acid, and 25 mM silver nitrate were first prepared, of which 5 mL of sodium citrate and 1 mL of tannic acid were added to 93 mL of water. The solution was then magnetically stirred at 500 rpm and heated to 100 °C. Once the solution was boiling, 1 mL of AgNO_3_ was added, and after 5 min, the heat was switched off and left to react for another 25 min. For the preparation of larger seeds, initial seeds were prepared using the above protocol, after which 50 mL of seed solution was added to 49 mL of a solution containing 5 mM sodium citrate and 25 μM tannic acid. The solution was then heated up to 90 °C for 10 min before adding 1 mL of 25 mM AgNO_3_ and left to react for 1 h. The process was repeated for each subsequent growth step to produce seeds of an increasing size.

### Synthesis of Ag–Au Nanoparticles Using CTAC and Ascorbic Acid

In a typical synthesis to prepare Ag_70_Au_30_NPs, a 5 mL solution containing 50 μM Ag (1 mL of original seed solution), 10 mM CTAC, and 2.14 mM ascorbic acid (AA) was prepared. While stirring at 25 °C, 10.7 μL of 10 mM tetrachloroaurate­(III) (HAuCl_4_) was added slowly to the Ag seed solution, resulting in a color change from yellow to red within 5 min. When preparing Ag–Au NPs of different Au percentage compositions, the molar ratios of Ag/CTAC and AA/Au were kept constant.

### Synthesis of Ag–Au Nanoparticles Using NaCl and HEPES

In a typical synthesis to prepare Ag_70_Au_30_NPs, a 5 mL solution containing 50 μM Ag (1 mL of original seed solution), 100 mM HEPES (pH 7), and 25 mM NaCl was prepared. While stirring at room temperature, 10.7 μL of 10 mM HAuCl_4_ was added slowly to the Ag seed solution, resulting in a color change from yellow to blue within 5 min. When preparing Ag–Au NPs of different Au percentages, the molar ratios of Ag/NaCl and HEPES/Au were kept constant.

## Results and Discussion

### Synthesis and Optical Characterization of Ag_70_Au_30_ Nanoparticles

The components required to synthesize Ag–Au NPs are Ag seeds, a capping agent, a reducing agent, and a Au precursor. As a guide, the key events of galvanic replacement, void formation, and reduction-assisted growth of Au and recovery of Ag are illustrated in Figure 1a. In this study, Ag NP seeds were prepared using sodium citrate and tannic acid, which were then used to explore two different approaches (Figure 1b). The first approach, termed the CTAC method, involved capping the Ag NPs with the surfactant cetyltrimethylammonium chloride (CTAC) and using ascorbic acid as the reducing agent. The CTAC concentration was optimized to ensure colloidal stability, as concentrations below 10 mM resulted in aggregation of Ag NPs (Figure S1).

**Figure 1:**
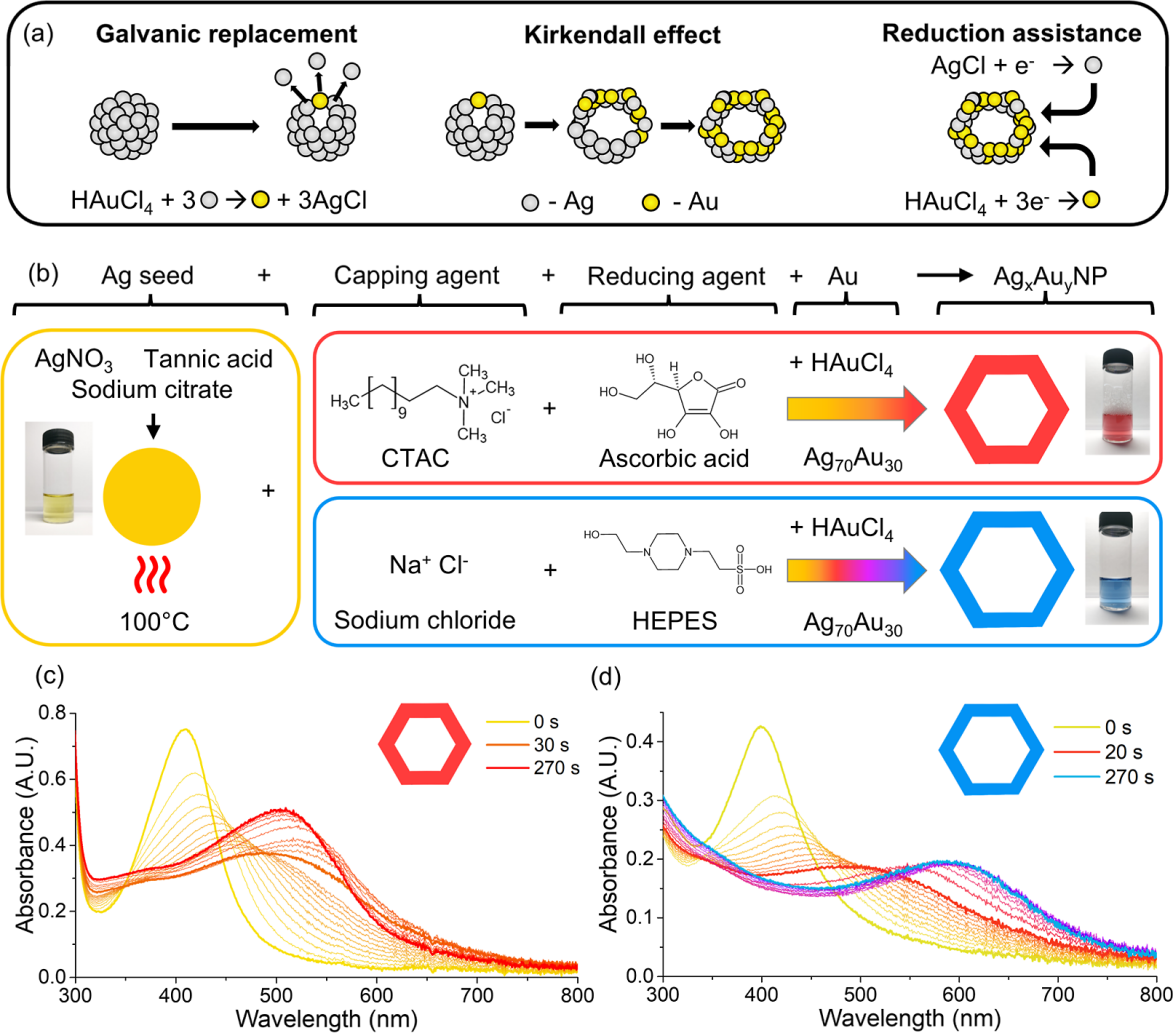
(a) Schematic representation of galvanic replacement, the Kirkendall effect, and reduction-assisted Au deposition and Ag recovery. (b) Schematic representation of the formation of Ag–Au NPs. (c) In situ absorbance scans during the formation of Ag_70_Au_30_ NPs using the CTAC method. (d) In situ absorbance scans during the formation of Ag_70_Au_30_ NPs using the NaCl method.

When 10 mM CTAC was employed, the LSPR peak exhibited a red shift from 405 to 408 nm, likely indicating a change in refractive index due to CTAC adsorption onto the nanoparticle surface. As a capping agent, CTAC has been shown to strongly cap the {100} facets of Ag NPs through the presence of excess Cl^–^ ions.^36,37^ In the absence of either a capping or reducing agent, the addition of HAuCl_4_ results in the complete disappearance of the Ag NP LSPR peak over time (Figure S2), consistent with full galvanic replacement. However, when CTAC is present, the intensity of the LSPR peak decreases but remains detectable, exhibiting a progressive red shift (Figure S3). This behavior is characteristic of hollow plasmonic nanoparticle formation,^30^ which can be modeled using Finite-Difference Time-Domain (FDTD) simulations of simple cuboctahedral structures (Figure S4). Simulations predict that increasing hollowness correlates with a greater red-shift in the LSPR peak (Figure S5).

Addition of a reducing agent provides an additional level of control over the synthesis by (i) reducing the HAuCl_4_ precursor, thereby mitigating the extent of galvanic replacement and (ii) restoring Ag lost during the replacement process. Ascorbic acid, a mild reducing agent, was selected to enable HAuCl_4_ reduction at room temperature without generating new Au seeds. We observed that higher ascorbic acid concentrations resulted in a blue-shift of the final LSPR peak (Figure S6), which was attributed to a combination of decreased hollowness (due to a decrease in the level of galvanic replacement) and increased Ag retention. Supporting this hypothesis, FDTD simulations showed that for hollow nanoparticles of a given size, higher Ag content produces a blue-shift in the LSPR (Figure S7).

Using 10 mM CTAC and a 100-fold molar excess of ascorbic acid with respect to Au, the formation of Ag_70_Au_30_ NPs was monitored over time using UV–vis spectroscopy (Figure 1c). It should be noted that Ag_*x*_Au_*y*_ denotes the relative Ag and Au contents and not the actual elemental composition of the final product. During the first 30 s, the LSPR peak intensity decreased while undergoing a noticeable red-shift (Figure 1c, orange line), after which the intensity began to recover alongside a slight blue-shift (Figure 1c, red line). This reversal likely reflects the reincorporation of Ag into the nanoparticle structure after complete reduction of the Au precursor resulting in less hollow and more Ag-rich architectures.

The second approach, termed the NaCl method, employed sodium chloride as a capping agent and HEPES (4-(2-hydroxyethyl)­piperazine-1-ethanesulfonic acid) as a reducing agent to investigate a surfactant-free approach. NaCl can interact with Ag surfaces to form AgCl, which we hypothesized would selectively protect {100} facets from galvanic replacement. HEPES, a Good’s buffer commonly used in biological applications, is also reported to act as a mild reducing agent for gold nanoparticle synthesis.^38,39^ Upon addition of NaCl to Ag NPs, the LSPR peak exhibited a blue-shift from 405 to 395 nm as the NaCl concentration increased to approximately 25 mM (Figure S8, red line), beyond which nanoparticle aggregation occurred (Figure S8, purple line). This blue-shift suggests modification of the Ag NP surface, likely due to the formation of AgCl. Notably, this shift proved critical for successful formation of Ag–Au NPs; samples with insufficient NaCl (<10 mM) did not result in the characteristic absorbance spectra of hollow Ag–Au NPs upon Au addition (Figure S9). While NaBr resulted in a similar LSPR blue-shift, it also caused a significant decrease in the absorbance (Figure S10). While HEPES alone caused no LSPR shift, in combination with NaCl, it produced a pronounced blue-shift accompanied by increased absorbance relative to NaCl alone, suggesting an additional role in enhancing colloidal stability (Figure S10). Similar to the CTAC-approach, NaCl alone was sufficient to generate hollow Ag–Au NPs, but the presence of HEPES yielded narrower LSPR bands at shorter wavelengths (Figure S11). However, the use of NaBr was less effective for Ag–Au NP formation (Figure S11) consistent with the literature reports that Cl^–^ ions are more effective than Br^–^ at capping {100} facets of Ag NPs. Similarly to ascorbic acid, increasing the HEPES concentration resulted in progressive blue-shifting of LSPR up to a certain point, beyond which additional HEPES primarily enhanced absorbance without further shifting (Figure S12). The NaCl-assisted synthesis did not exclusively rely on HEPES as the reducing agent, with 25 μM ascorbic acid also producing results similar to those of 100 mM HEPES (Figure S13). Using 25 mM NaCl and 100 mM HEPES, we monitored the formation of Ag_70_Au_30_NPs over time using UV–vis spectroscopy (Figure 1d). Compared to the CTAC approach, the LSPR in the NaCl methods evolved more rapidly and exhibited a larger red-shift (Figure S14), resulting in a blue dispersion rather than a red. These observations suggest that Ag_70_Au_30_ NPs formed using the NaCl method are more hollow than those produced by using the CTAC method.

To gain deeper insight into the morphology of Ag_70_Au_30_ NPs, TEM and HAADF-STEM were used to obtain both the structural images and elemental mapping information. The AgNP seeds were found to be 15.5 ± 1.9 nm in diameter (Figure S15). Both Ag_70_Au_30_ NP samples synthesized via the CTAC and NaCl methods appeared as hollow hexagonal structures under TEM (Figure 2). We attribute this morphology to the selective capping of {100} facets by CTAC and NaCl, which likely promotes galvanic replacement and subsequent growth along the {111} facets. The presence of {111} facets was confirmed through the atomic lattice spacing measurements, revealing characteristic interplanar *d*-spacings of 0.238 nm for Ag seeds and 0.245 and 0.239 nm for Ag_70_Au_30_NP (CTAC) and Ag_70_Au_30_NP (NaCl), respectively (Figures S16–S18), consistent with the {111} planes of face-centered cubic (fcc) structures. The elemental distribution was then explored using EDX, indicating that Ag and Au were homogeneously distributed throughout the nanoparticles rather than forming a core@shell configuration (Figures 2 and S19–S21). Using EDX atomic fractions were determined to be 71% Ag and 29% Au for Ag_70_Au_30_NP (CTAC) and 68% Ag and 32% Au for Ag_70_Au_30_NP (NaCl). These measurements closely match the values of 70% Ag and 30% Au, indicating minimal silver loss during the synthesis.

**Figure 2:**
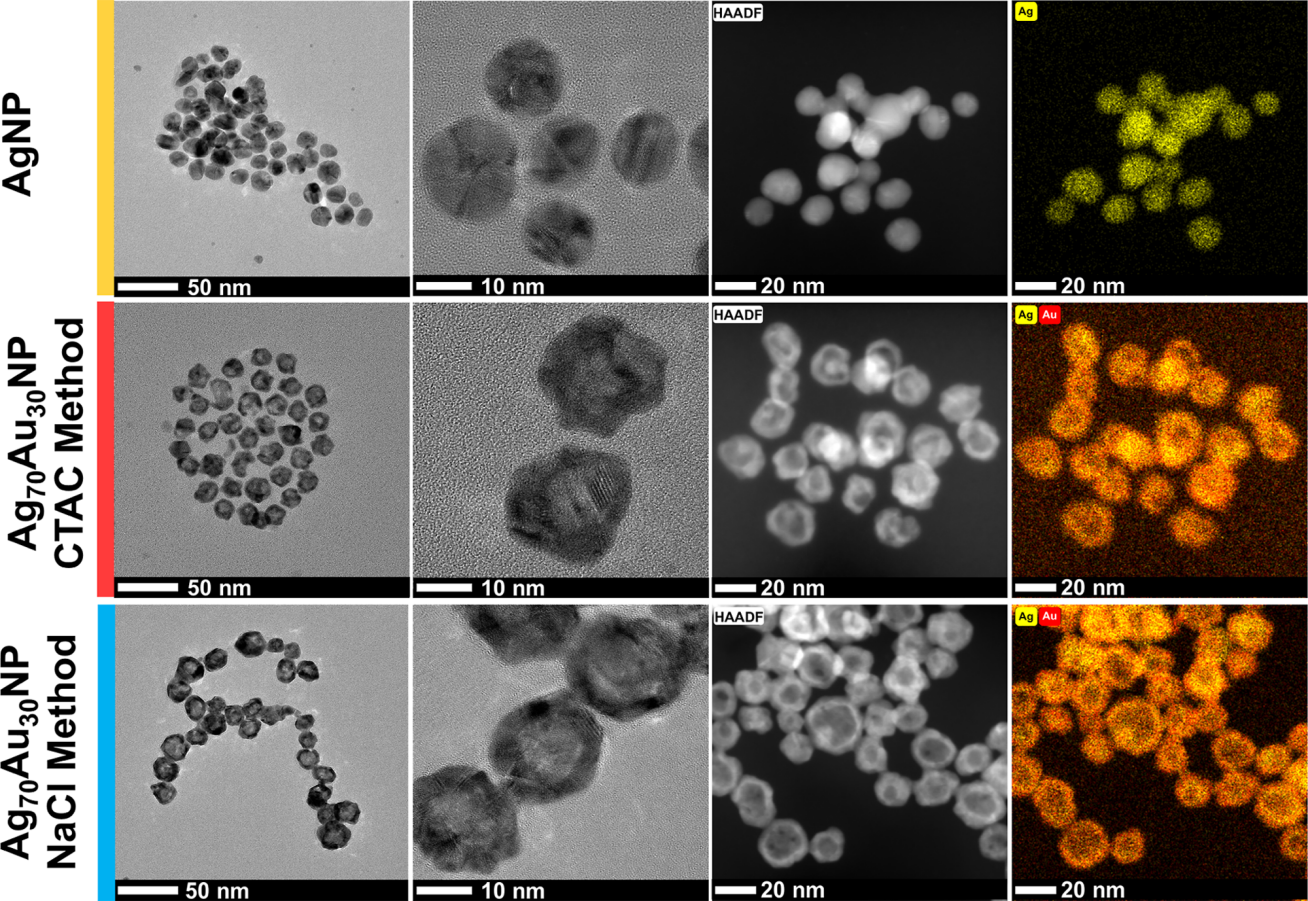
TEM, HAADF-STEM, and elemental mapping images of AgNP, Ag_70_Au_30_NP (CTAC), and Ag_70_Au_30_NP (NaCl).

### Temperature-Induced Morphology Changes

Although Ag_70_Au_30_ NPs produced via both methods were colloidally stable, their optical properties evolved over time with the color of the dispersions changing from red/blue to orange/yellow over several days to weeks. This change was significantly accelerated by elevated temperature, with complete color change observed within 8 h at 70 °C (ESI, Figures S22 and S23). While others have reported that this blue-shift can be driven by the reincorporation of Ag into the Ag–Au NPs,^40^ our results suggest that most Ag recovery occurs prior to this stage. We therefore hypothesize that the observed spectral evolution primarily results from the structural collapse of the hollow core into a more compact, solid nanoparticle.

To investigate whether the hollow core morphology can be preserved, we examined the effect of an additional strong capping agent, glutathione (GSH), to arrest the structural transformation (Figure S24). Adding excess GSH decreased the colloidal stability; however, a concentration of 10 μM GSH was found to be sufficient for stabilization (Figure S25). After heating at 70 °C for 8 h, distinct color changes were observed. Ag_70_Au_30_ NP (CTAC) turned orange, Ag_70_Au_30_NP (NaCl) turned yellow, and Ag_70_Au_30_ NP (NaCl + GSH) turned pink (Figure 3a). The corresponding normalized absorbance spectra (Figure 3b) revealed significant overlap between the heated Ag_70_Au_30_ NP (CTAC) and Ag_70_Au_30_ NP (NaCl) samples, while Ag_70_Au_30_ NP (NaCl + GSH) was notably red-shifted from the rest. All heated nanoparticles exhibited good long-term stability over 6 weeks, with only minor spectral changes observed during this period (Figures S26–S28).

**Figure 3:**
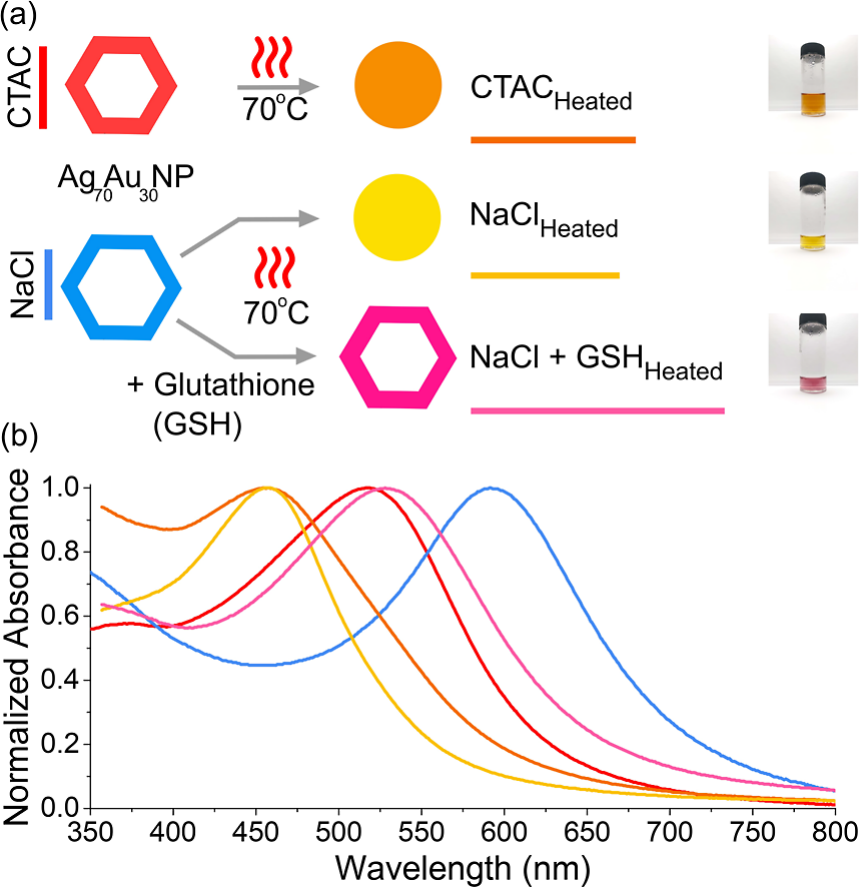
(a) Schematic representation of the heat-induced morphology change, along with photographs of the resulting samples. (b) Normalized absorbance spectra of each of the samples depicted in (a).

TEM images showed that the majority of heated Ag_70_Au_30_ NPs without GSH became solid (Figure 4), with diameters of 17.7 ± 1.9 and 17.6 ± 2.5 for CTAC and NaCl-route, respectively (Figures S29 and S30).

**Figure 4:**
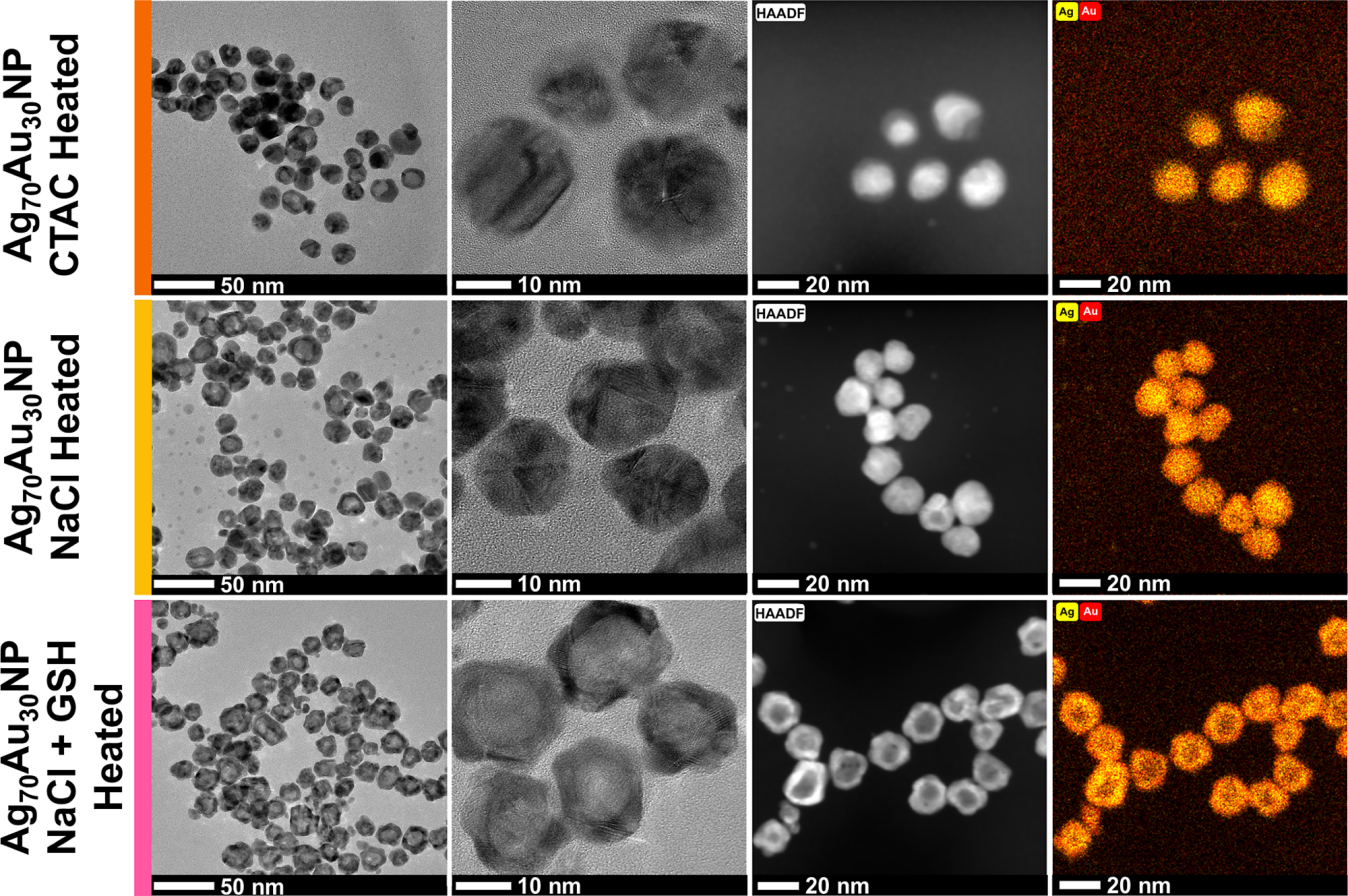
TEM, HAADF, and elemental mapping images of Ag_70_Au_30_NP (CTAC), Ag_70_Au_30_NP (NaCl), and Ag_70_Au_30_NP (NaCl + GSH) after heating at 70 °C for 6 h.

Notably, both synthetic approaches resulted in nanoparticles with comparable optical and size properties. In addition, their larger diameters relative to the Ag seeds provided additional evidence that minimal Ag loss occurs during alloy formation. The absorbance spectra closely matched the simulated spectra for an 18 nm Ag_70_Au_30_ NPs (Figure S31), supporting the structural consistency of the synthesized particles. In contrast to thermally treated NPs prepared without GSH, the majority of the heated Ag_70_Au_30_ NPs (NaCl + GSH) retained their hollow shape rather than collapsing into solid structures (Figure 4). Stabilization is likely due to the presence of the sulfhydryl (-SH) group in GSH, which forms strong Ag–S or Au–S bonds with surface atoms. Such interactions can inhibit the migration of these atoms, thereby preserving the hollow core, even after thermal treatment. This introduces an additional level of control in Ag–Au NP synthesis, enabling nanoparticles synthesized from identical Ag seeds and with identical elemental composition to exhibit distinct plasmonic properties, depending on how hollow they are designed to be. In all cases, EDX analysis confirmed homogeneous alloying of Ag and Au throughout the nanoparticles (Figures 4 and S32–S34).

### Synthesis of Ag–Au NP across a Range of Au Percentages

The elemental composition of Ag–Au NPs plays a critical role in determining their plasmonic properties. Therefore, precise control over this parameter is essential for a tailored Ag–Au NP synthesis. Using both CTAC and NaCl methods, Ag–Au NPs were prepared with varying amounts of Au and subsequently heated overnight to form stable alloy nanoparticles (Figure 5a). Corresponding photographs and absorbance spectra of all samples, both pre- and postheating, are provided in ESI (Figures S35 and S36).

**Figure 5:**
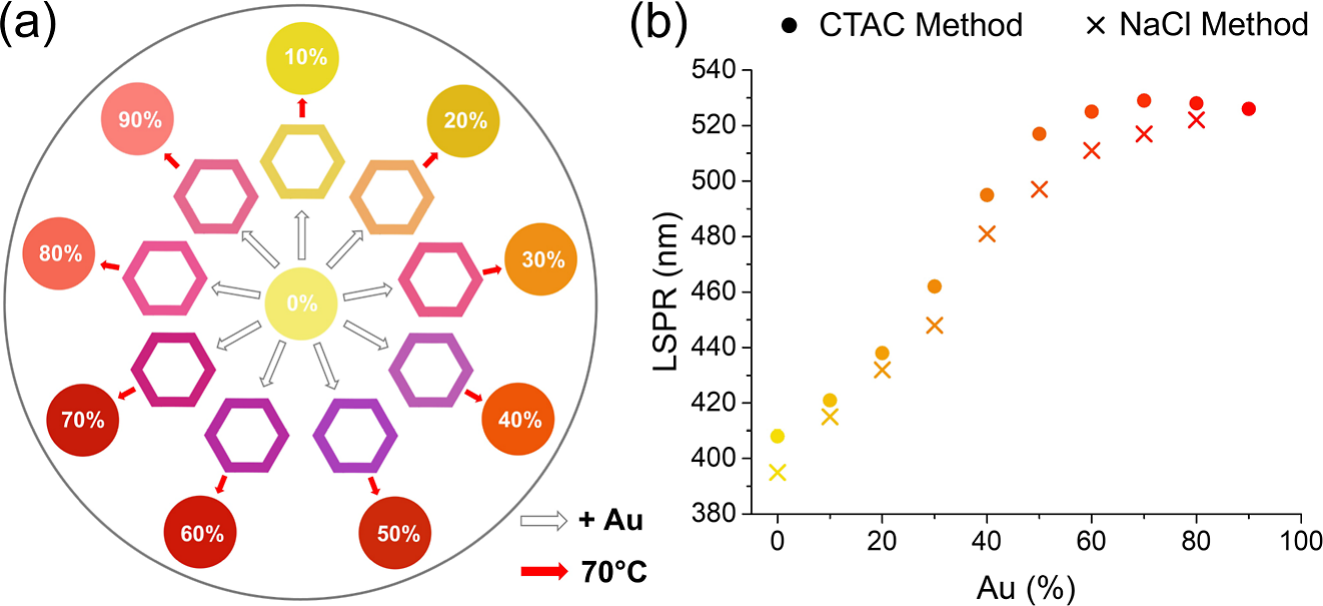
(a) Schematic showing the effect of addition of different Au percentages, followed by subsequent heating at 70 °C overnight. (b) The change in LSPR at different Au percentages for alloys made using both the CTAC and NaCl method after heating.

For solid Ag–Au NPs, the LSPR wavelengths are generally expected to increase linearly with the Au content. This trend was observed at lower Au percentages (Figure 5b); however, beyond ∼30% Au, a pronounced red-shift in the LSPR occurred, after which the peak position gradually converged at higher Au percentage. This suggests that at a higher percentage of Au, the rate of galvanic replacement is not adequately controlled, leading to an increased loss of Ag and a higher-than-intended Au percentage in the final product.

While future strategies, such as multistep Au addition over a longer period, could mitigate this effect, a practical alternative is to adjust the initial Au input to achieve the desired final composition. It should be noted that while the CTAC-based method consistently produced colloidally stable nanoparticles across the whole compositional range, the NaCl method failed to maintain stability at high Au content (e.g., Ag_10_Au_90_) Nevertheless, these results demonstrate that both approaches can generate stable alloy nanoparticles with tunable LSPR between 395 and 520 nm.

### Increasing Ag Seed Size Promotes Further Red-Shift in Ag–Au NPs

Another benefit of seed-based approaches is the ability to tune the size of the resulting Ag–Au NPs by independently adjusting the size of the AgNP seeds. To investigate this, we examined how both synthesis methods respond to different AgNP seed sizes. Larger Ag seeds were expected to yield larger hollow Ag–Au NPs, leading to a further red-shift of the LSPR toward the NIR region. AgNP seeds with diameters of 20, 27, 34, 44, 55, and 71 nm were prepared through successive Ag growth steps and subsequently used to make Ag_70_Au_30_NP (Figure 6a). TEM images and size distribution analyses of the Ag seeds are provided in ESI (Figures S37 and S38).

**Figure 6:**
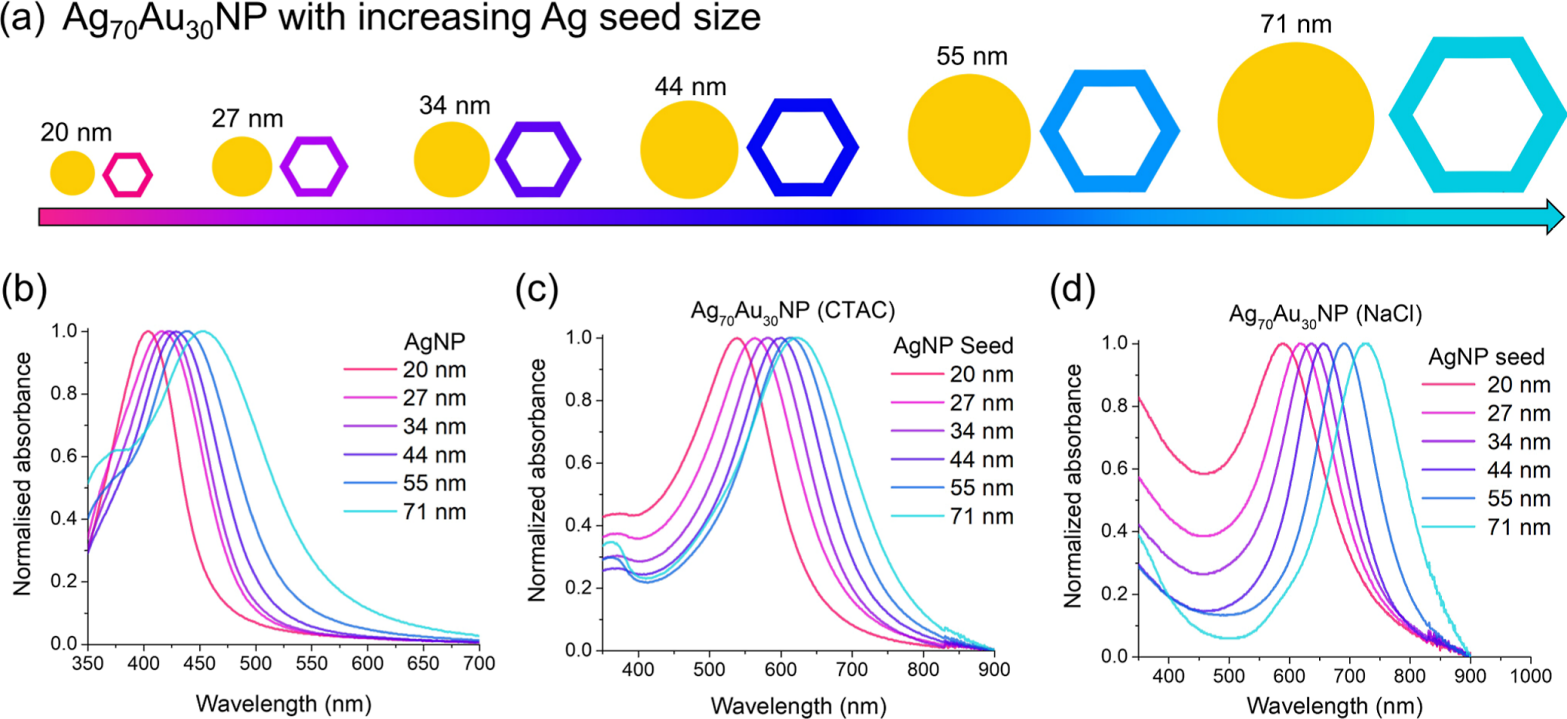
(a) Schematic showing the relationship between increasing seed size and absorbance properties of the resulting alloy nanoparticles. (b) Absorbance spectra of AgNPs of different sizes used to make Ag–Au NPs. (c) Absorbance spectra of Ag_70_Au_30_NP made from each of the AgNP seeds using the CTAC method. (d) Absorbance spectra of Ag_70_Au_30_NP made from each of the AgNP seeds using the NaCl method.

As expected, the LSPR of the Ag seeds (Figure 6b) exhibited a red-shift with increasing seed size.^35^ Producing Ag_70_Au_30_ NPs from these seeds resulted in a gradual increase in the LSPR wavelength as the seed size increased, with NPs made via the NaCl method consistently showing higher LSPR values than those prepared using CTAC, reaching a maximum of 726 nm (Figure 6b,c).

Interestingly, the seed size also affected the thermal behavior of the Ag_70_Au_30_ NPs. For Ag_70_Au_30_ NPs synthesized via the CTAC method, larger seeds exhibited smaller LSPR shifts after heating (Figure S39). This suggests that there is a size threshold above which Ag–Au NPs do not fully collapse but instead retain a hollow structure. The effect of GSH addition prior to heating was also explored (Figure S40a–c). For Ag_70_Au_30_NPs prepared with seeds smaller than 44 nm, the presence of 10 μM GSH reduced the LSPR change compared to samples without GSH (Figure S40d). Conversely, Ag_70_Au_30_NPs made using 55 and 71 nm seeds showed an increase in LSPR after heating, with GSH inducing a slightly larger increase (Figure S40d). This indicates that GSH can modulate the morphology of Ag–Au NPs (CTAC method) prepared using smaller seeds, but its effect diminishes with increasing seed size. Notably, all CTCA-derived Ag_70_Au_30_ NPs remained colloidally stable after heating, in contrast to those synthesized by using the NaCl method. Heating NaCl-derived Ag_70_Au_30_ NPs caused a much greater LSPR shift, and NPs made using 55 and 71 nm seeds completely lost their color after heating (Figure S39). To further elucidate GSH’s role, we monitored LSPR changes during heating of Ag_70_Au_30_NPs (NaCl method) at varying GSH concentrations (Figure 7).

**Figure 7:**
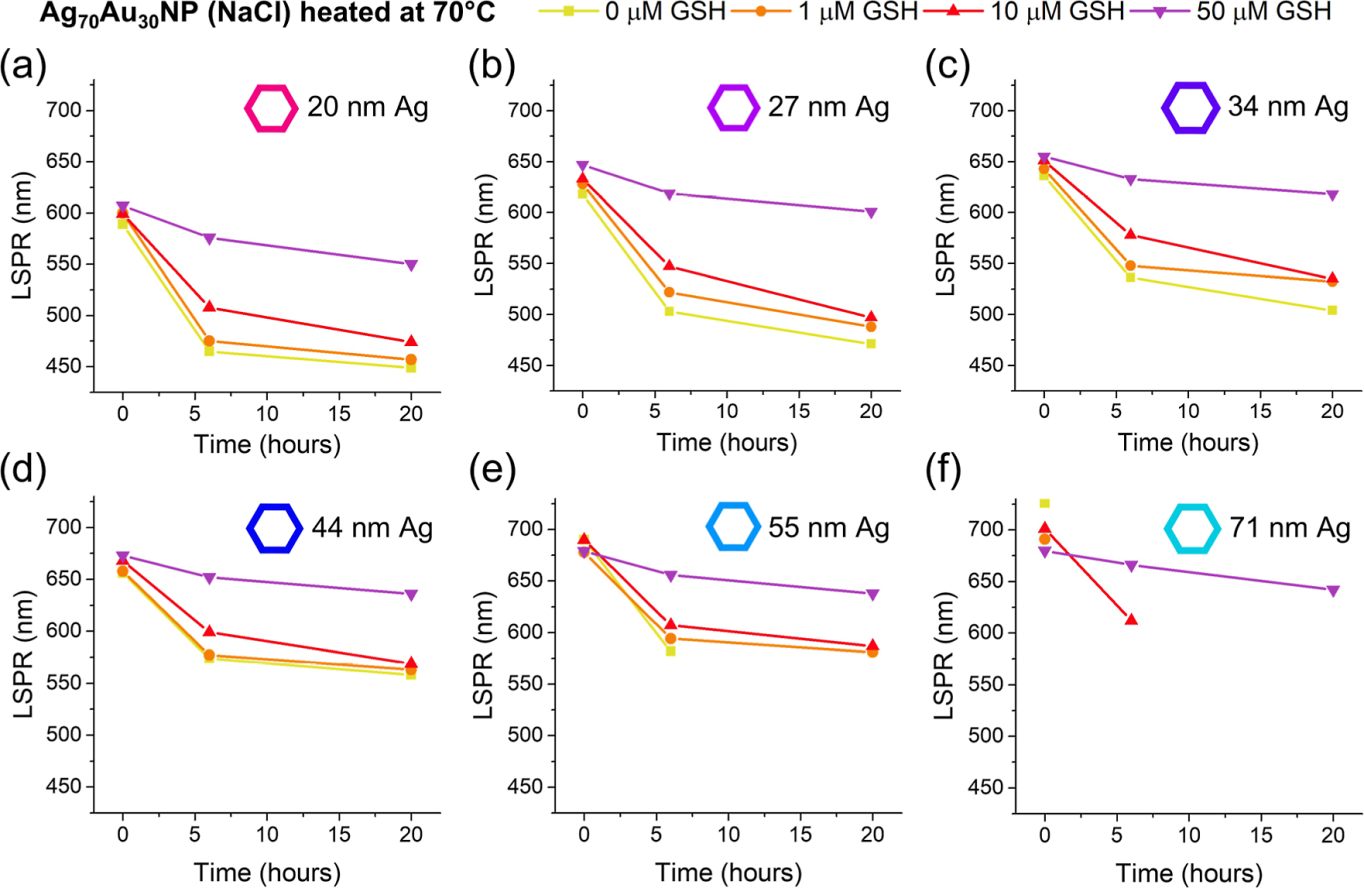
Shift in LSPR over time with Ag_70_Au_30_NP (NaCl) heated with different concentrations of GSH made using (a) 20, (b) 27, (c) 34, (d) 44, (e) 55, and (f) 71 nm sized AgNP seeds.

Across all samples, higher GSH concentrations consistently reduced the LSPR shifts during heating. Importantly, Ag_70_Au_30_NPs prepared with 55 and 71 nm seeds only remained stable postheating when 50 μM GSH was present. Full absorbance spectra are provided in the ESI (Figure S41a–d). Overall, these findings highlight GSH as a key stabilizer for larger Ag–Au NPs, offering an alternative to strong surfactants.

## Conclusion

Two distinct approaches for the synthesis of Ag–Au alloy nanoparticles from Ag seeds have been developed, highlighting the critical roles of both capping and reducing agents in determining the final morphology and, consequently, the plasmonic properties of resulting nanostructures. The choice of the capping agent was found to be crucial in facilitating the formation of Ag–Au NPs through the selective protection of Ag {100} facets, while the strength of the reducing agent can tune the degree of hollowness and Ag recovery. Postsynthetic heating accelerated structural transformation toward thermodynamically stable structures, often resulting in reduced hollowness or collapsed hollow core, a process influenced by seed size and the presence of GSH. The use of GSH was found to be a highly effective tool for increasing the stability of hollow Ag–Au NPs prepared using the NaCl-based approach, which marks another significant advance in reducing the reliance on surfactants. Both synthetic approaches offer broad tunability in terms of elemental composition and particle size, allowing for the precise adjustment of localized surface plasmon resonance (LSPR) across the visible spectrum. While the CTAC-based method provides superior colloidal stability at higher Au content and larger seed sizes, the NaCl-based approach can be used as a robust, surfactant-free alternative for applications where the use of surfactants is undesirable, such as in hybrid photocatalyst design. The presented systematic investigation into nanoelectronic particles serves as a practical framework for tailoring Ag–Au NPs to meet specific structural and optical requirements for future applications ranging from sensor design to advanced catalysis.

## Supplementary Material



## Notes

Methodology of the computational simulations used within this work, along with supplementary figures (PDF)

## Abbreviations

AA: ascorbic acid
AgNP: silver nanoparticle
Ag–Au NP: silver–gold nanoparticle
AuNP: gold nanoparticle
CTAC: hexadecyltrimethylammonium chloride
EDX: energy-dispersive X-ray spectroscopy
FDTD: frequency-difference time-domain
GSH: glutathione
HAADF-STEM: high-angle annular dark-field scanning transmission electron microscopy
HEPES: (4-(2-hydroxyethyl)-1-piperazineethanesulfonic acid)
LSPR: localized surface plasmon resonance
SERS: surface-enhanced Raman spectroscopy
TEM: transmission electron microscopy.
