# Supplementary material for: Nanoelectrum across the Visible Spectrum: Tailored Ag–Au Alloy Nanoparticles with Glutathione-Enhanced Stability
Source: Chem Mater. 2025 Dec 6;38(1):181–9. doi: 10.1021/acs.chemmater.5c02141 (PMC12805512; doi:10.1021/acs.chemmater.5c02141)
Supplement: Supplementary file 1 [file cm5c02141_si_001.pdf]

Nanoelectrum Across the Visible Spectrum: Tailored Ag-Au Alloy Nanoparticles with Glutathione-enhanced Stability

Electronic Supplementary Information

Matthew G. Ellis[1]*, Oriol Colomer I Ferrer[2,3], Muhamad Hartono[2], Olga Niarchou[2], Ali Zarkesh[2], Tijmen G. Euser[1], Ljiljana Fruk[2]*.

1. Nano Photonics Centre, Cavendish Laboratory, Department of Physics, University of Cambridge, JJ Thomson Ave, Cambridge CB3 0HE, UK

2. Department of Chemical Engineering and Biotechnology, University of Cambridge, Phillipa Fawcett Drive, Cambridge CB3 0AS, UK

3. Hitachi Cambridge Laboratory, Hitachi Europe Ltd, J. J. Thomson Avenue, Cambridge CB3 0HE, UK

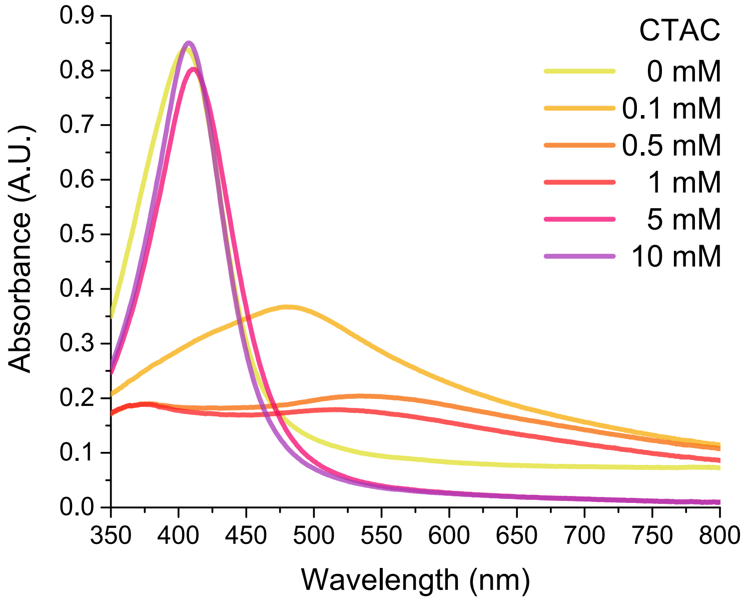

Figure S1. Absorbance spectra of AgNP (16 nm) mixed with different concentrations of CTAC.

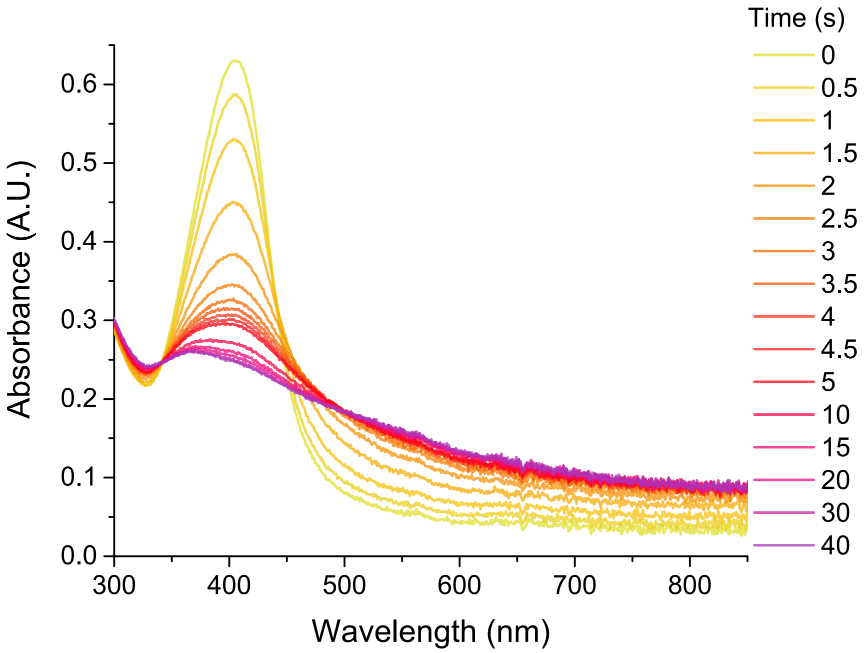

Figure S2. In situ absorbance scans during the formation of $Ag_{70}Au_{30}NP$ without the presence of any additional capping or reducing agent (direct addition of $HAuCl_4$ to AgNP seeds).

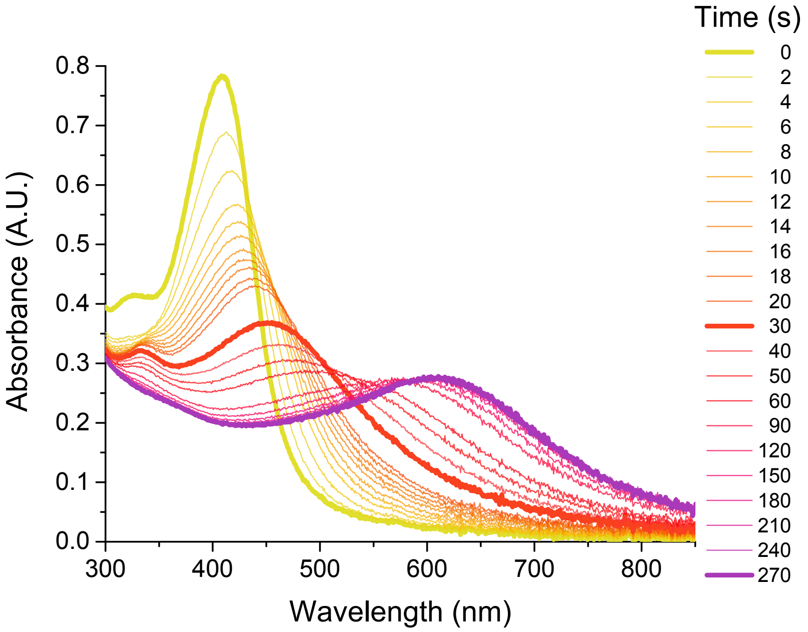

Figure S3. In situ absorbance scans during the formation of $Ag_{70}Au_{30}NP$ in the presence of 10 mM CTAC (no reducing agent).

FDTD methods

Computational work to simulate nanoparticle absorbance properties was carried out using a finite-difference time-domain (FDTD) method (Lumerical Solutions). A mesh size of 75 nm x 75 nm x 75 nm was used with a max mesh step of 1 nm. A total-field scattered-field (TFSF) light source (200 – 700 nm) was used. Solid nanoparticles were modelled using spheres while cuboctahedron shapes were used to model the hollow nanoparticles. A background refractive index of 1.333 was used for all measurements. Palik (0 – 2 μm) was used for the refractive index of Ag, while the refractive indexes of Ag/Au alloy materials were obtained using Refreactiveindex.info,[1] which used data reported by Rioux *et al.* (2014).[2]

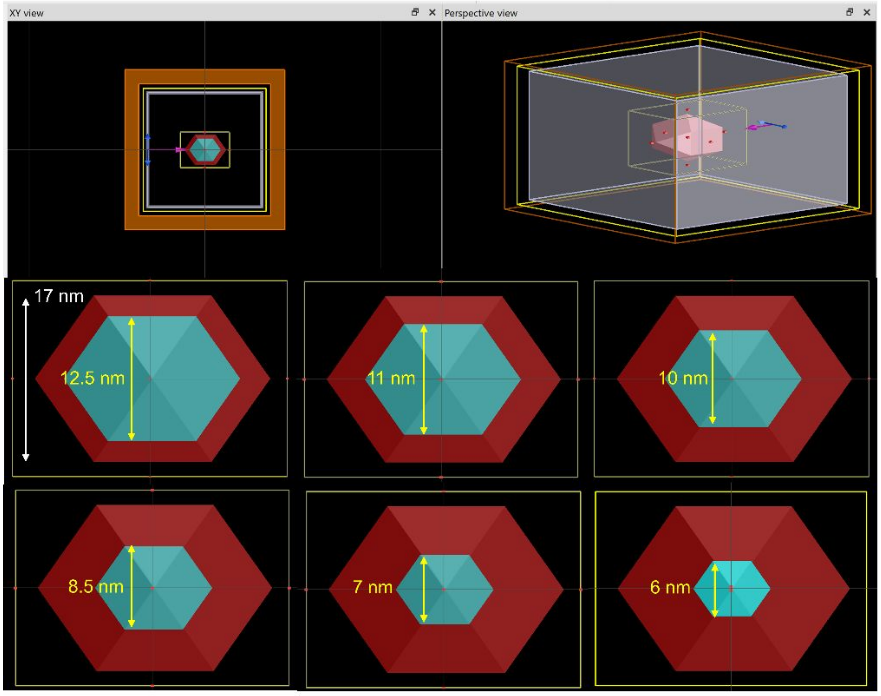

Figure S4. Lumerical FDTD models used to simulate the absorbance properties of $Ag_{70}Au_{30}NP$ with different sized hollow cores.

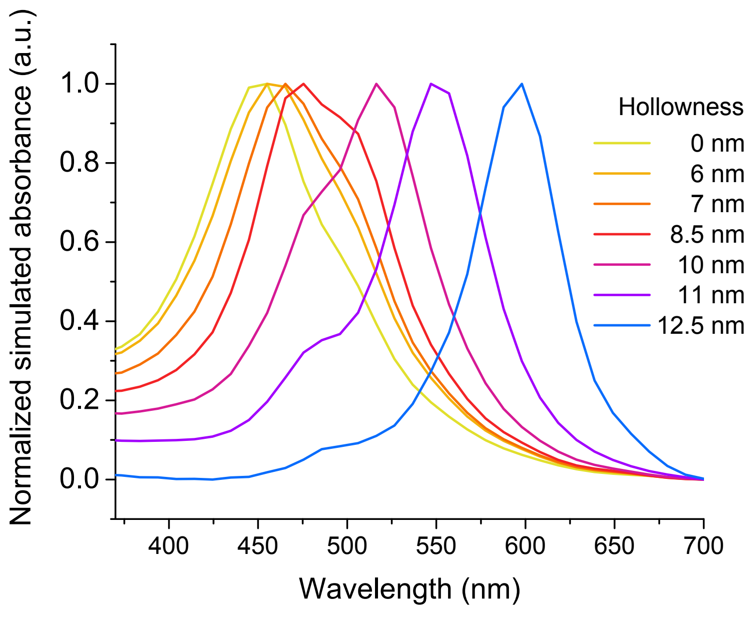

Figure S5. Simulated absorbance spectra $Ag_{70}Au_{30}$NP with different sized hollow cores.

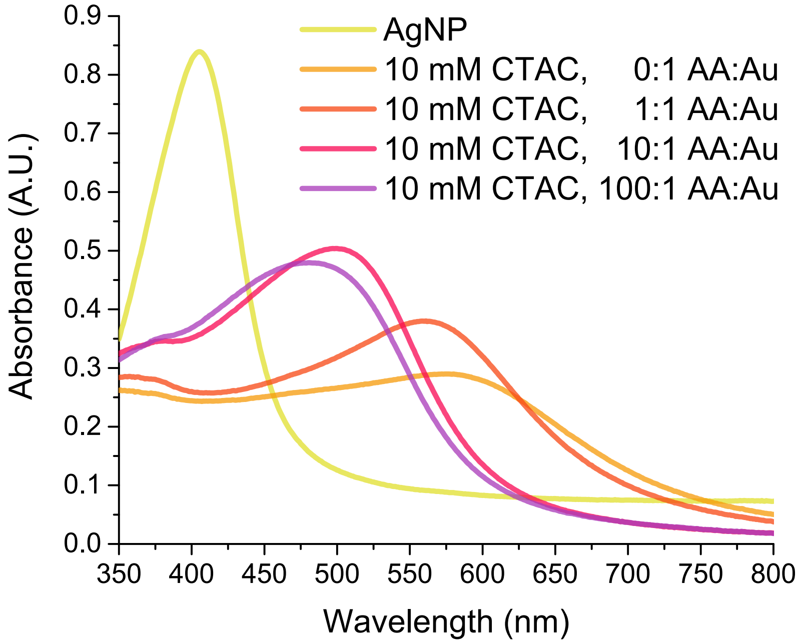

Figure S6. Absorbance spectra of $Ag_{70}Au_{30}$NP made using 10 mM CTAC and varying Molar ratios of ascorbic acid relative to Au. Spectra taken 30 minutes after synthesis.

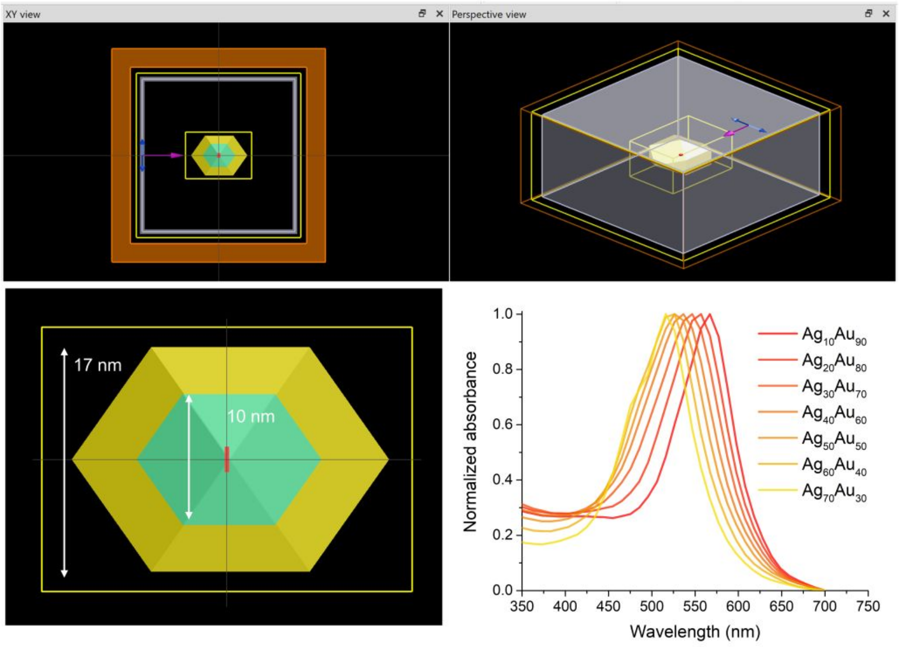

Figure S7. Lumerical FDTD simulation model of the absorbance properties of hollow AgAuNP with different elemental compositions.

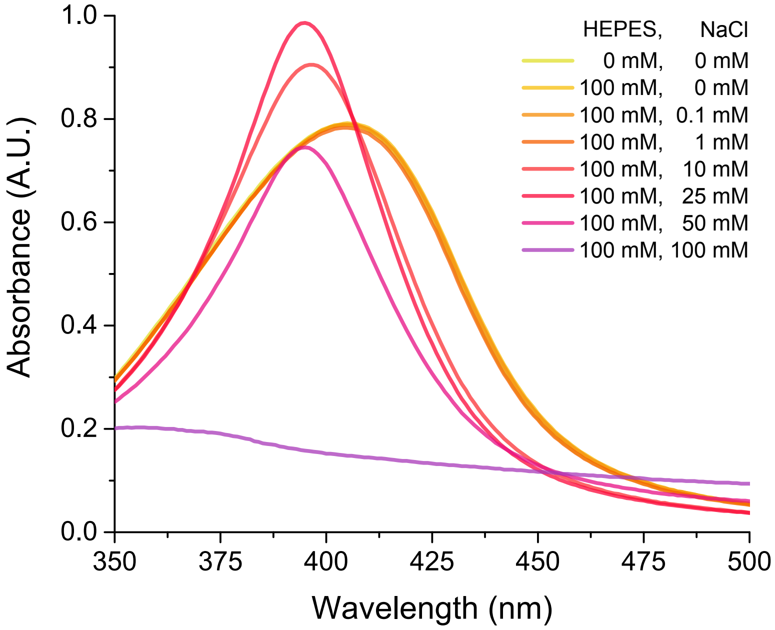

Figure S8. Absorbance spectra of AgNP mixed with different amounts of HEPES and NaCl. Spectra taken 30 minutes after synthesis.

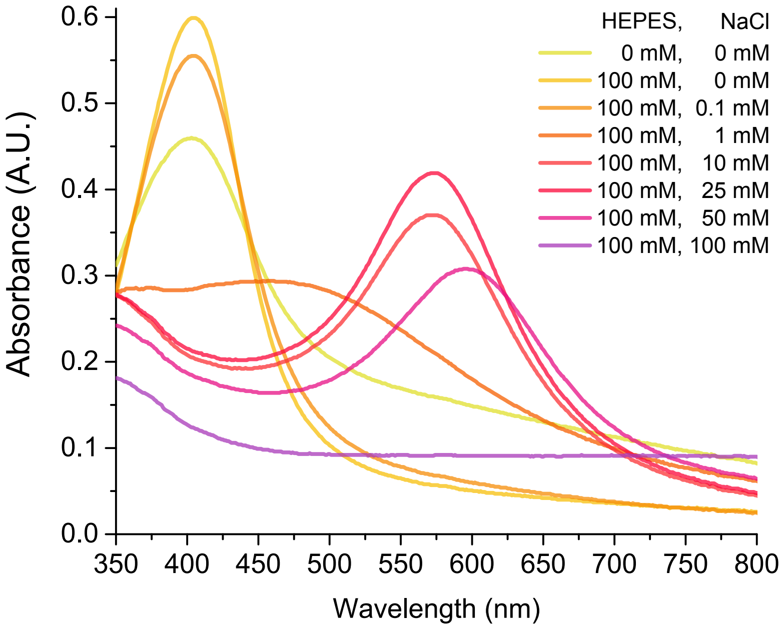

Figure S9. Absorbance spectra of $Ag_{70}Au_{30}NP$ made by adding $HAuCl_4$ to AgNP mixed with different amounts of HEPES and NaCl. Spectra taken 30 minutes after synthesis.

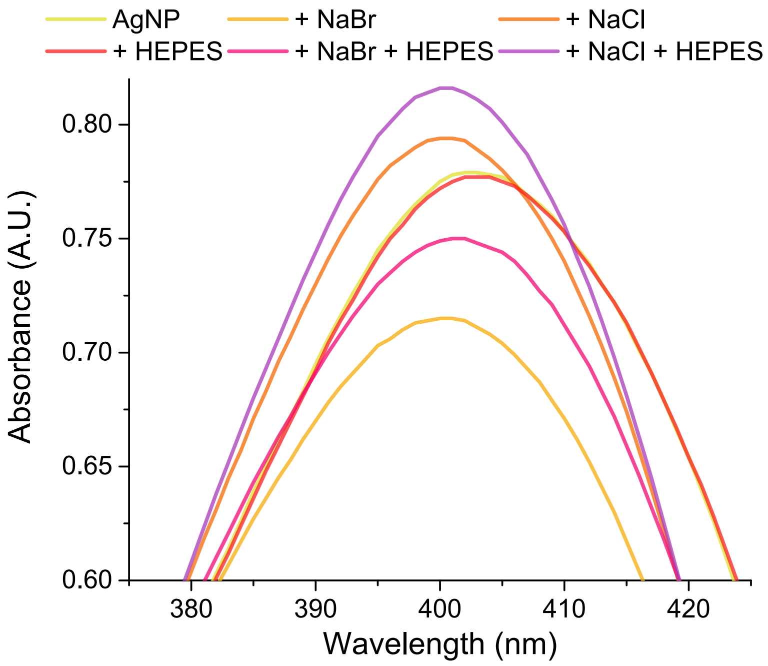

Figure S10. Absorbance spectra of AgNP mixed with either 25 mM NaBr, 25 mM NaCl, 100 mM HEPES, 25 mM NaBr with 100 mM HEPES, and 25 mM NaCl with 100 mM HEPES. Spectra taken 30 minutes after synthesis.

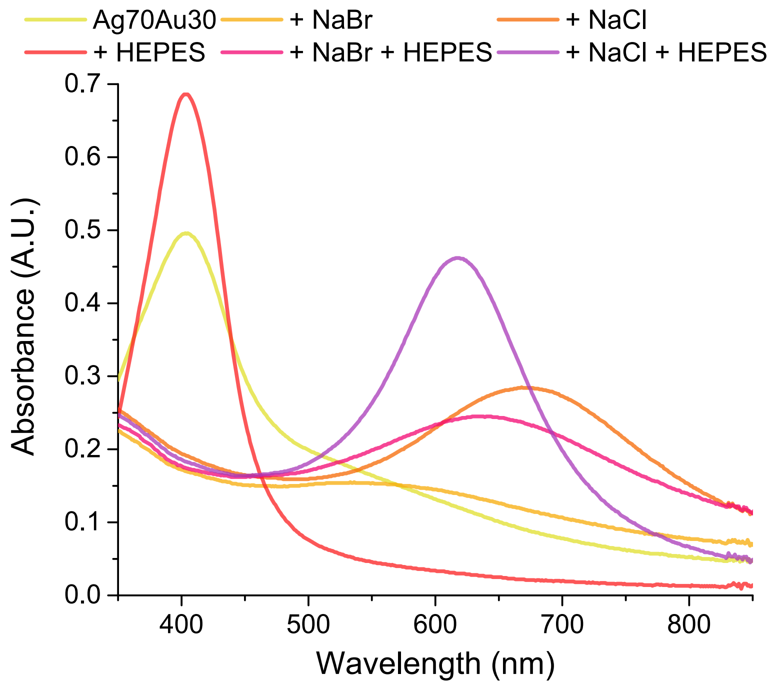

Figure S11. Absorbance spectra of $Ag_{70}Au_{30}$NP made by adding $HAuCl_4$ to the samples shown within Figure S10. Spectra taken 30 minutes after synthesis.

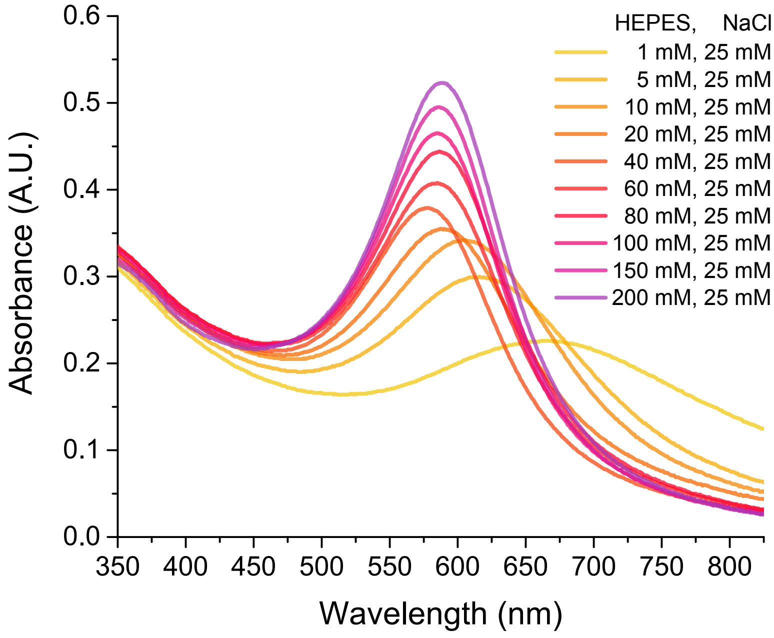

Figure S12. Absorbance spectra of $Ag_{70}Au_{30}$NP made using 25 mM NaCl and varying amounts of HEPES buffer. Spectra taken 30 minutes after synthesis.

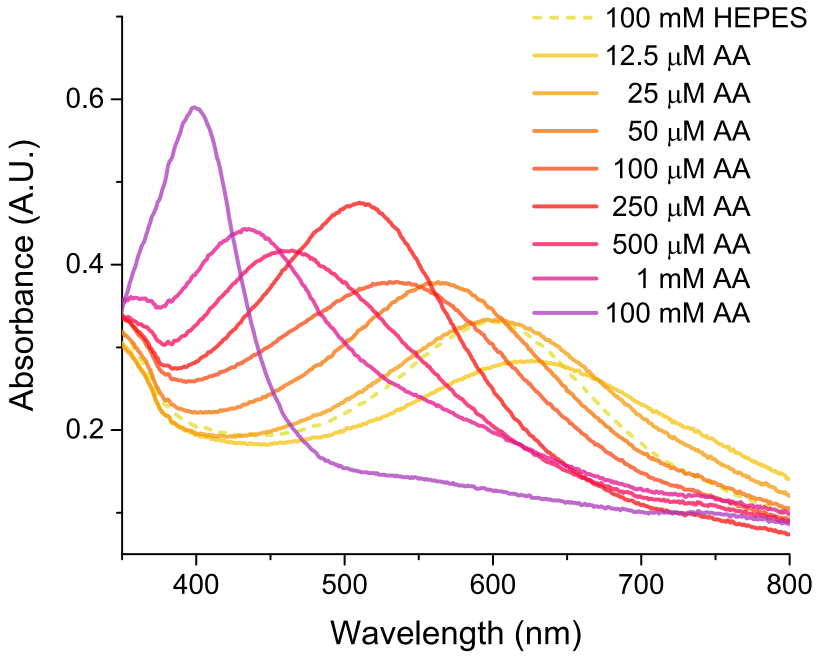

Figure S13. Absorbance spectra of $Ag_{70}Au_{30}$NP made using 25 mM NaCl, with 100 mM HEPES and different concentrations of ascorbic acid (AA). Spectra taken 30 minutes after synthesis.

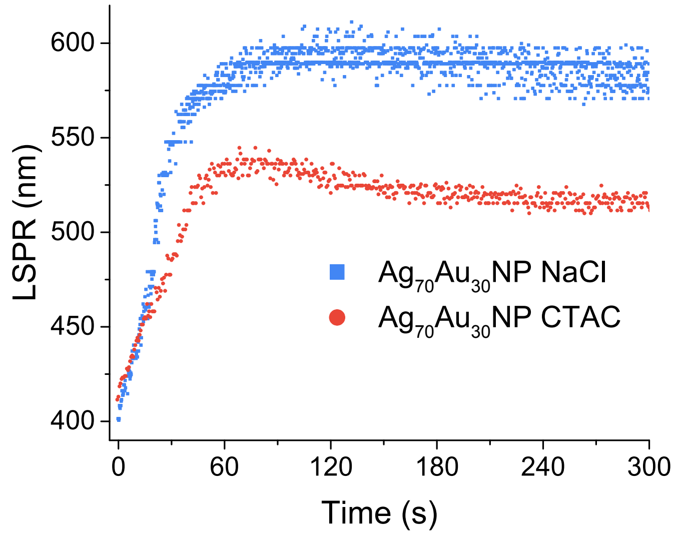

Figure S14. Change in peak LSPR position over time during the synthesis of $Ag_{70}Au_{30}$NP (NaCl and CTAC method).

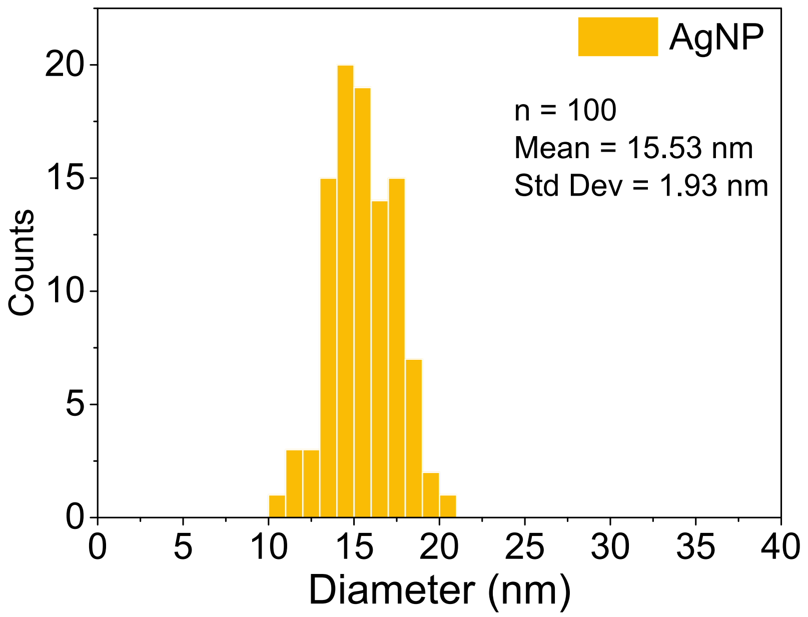

Figure S15. Size distribution of the initial AgNP seeds used within this work.

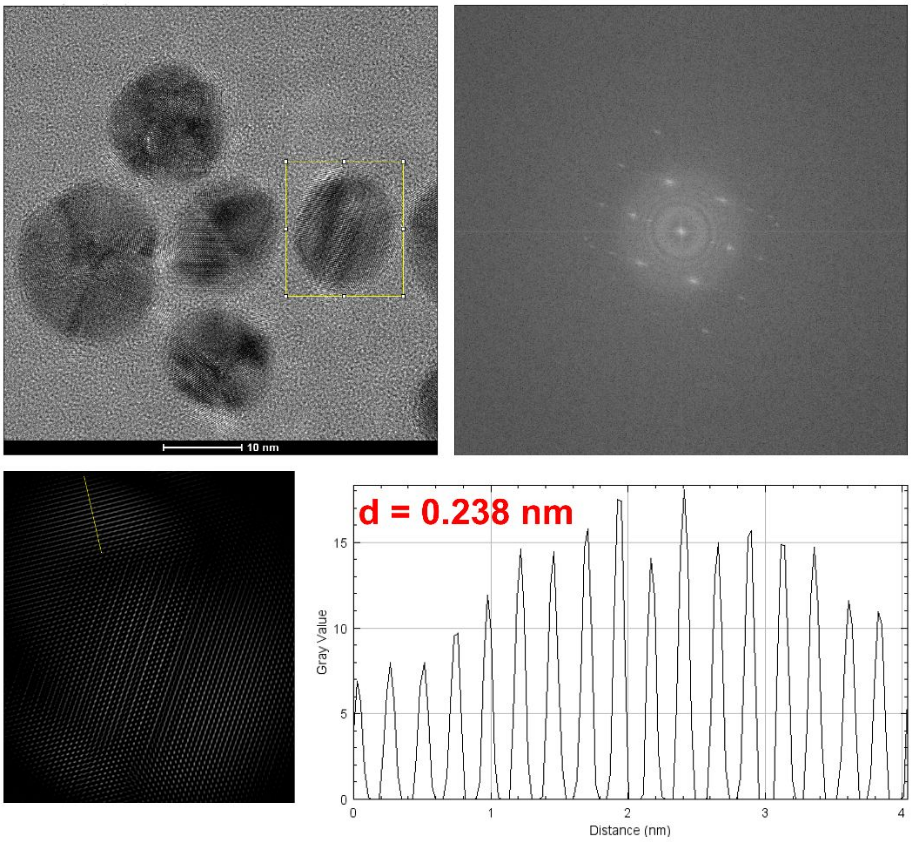

Figure S16. Use of ImageJ to derive d-spacing of AgNP

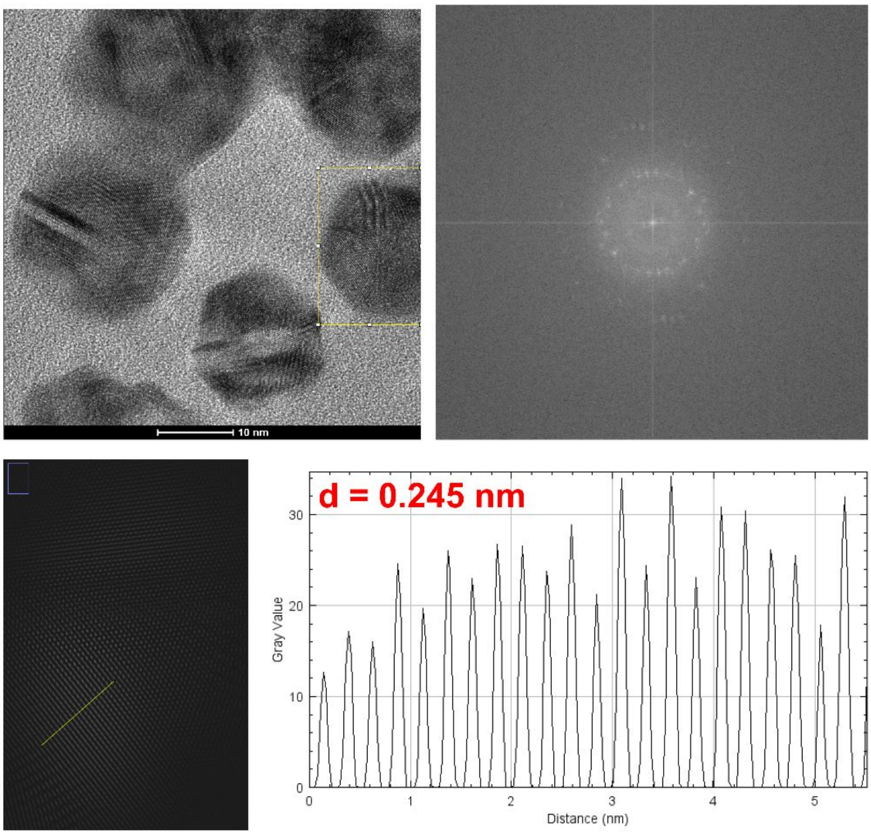

Figure S17. Use of ImageJ to derive d-spacing of $Ag_{70}Au_{30}$NP CTAC method.

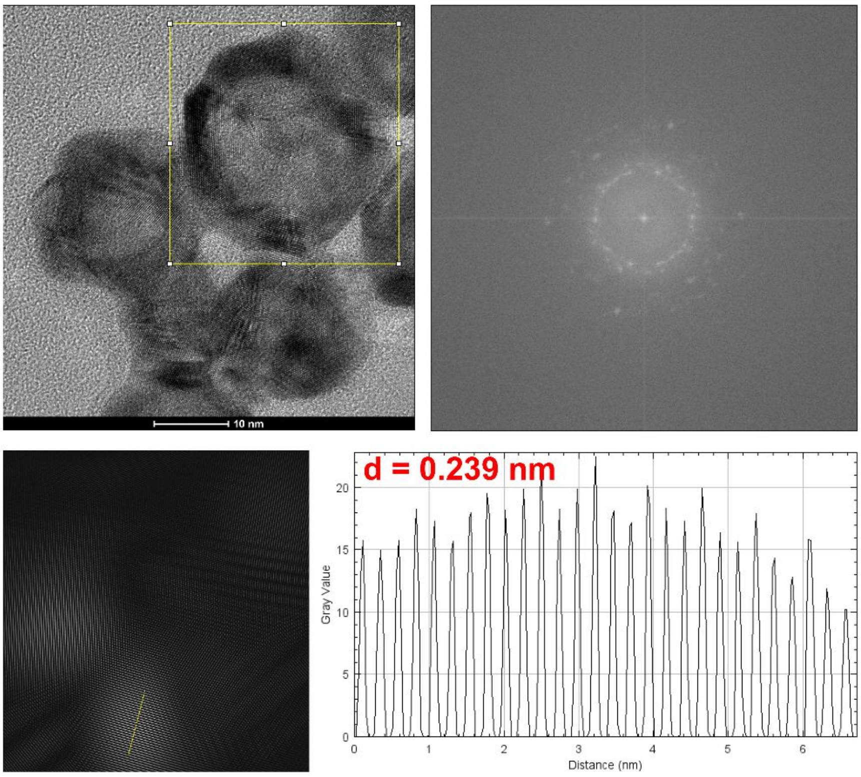

Figure S18. Use of ImageJ to derive d-spacing of $Ag_{70}Au_{30}$NP NaCl.

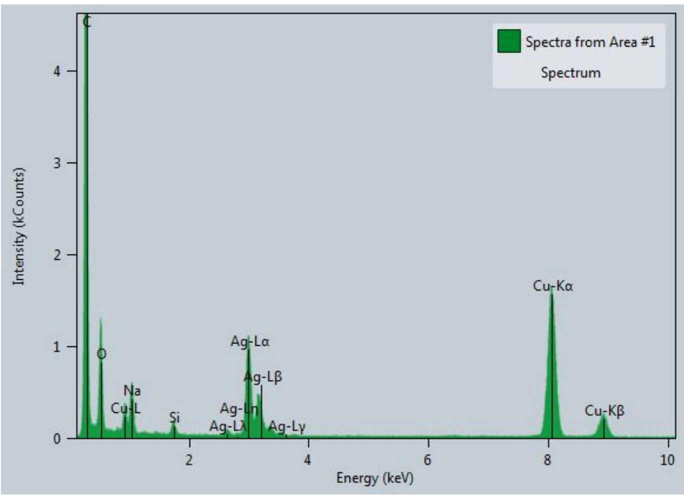

Figure S19. EDX spectrum of the AgNP sample.

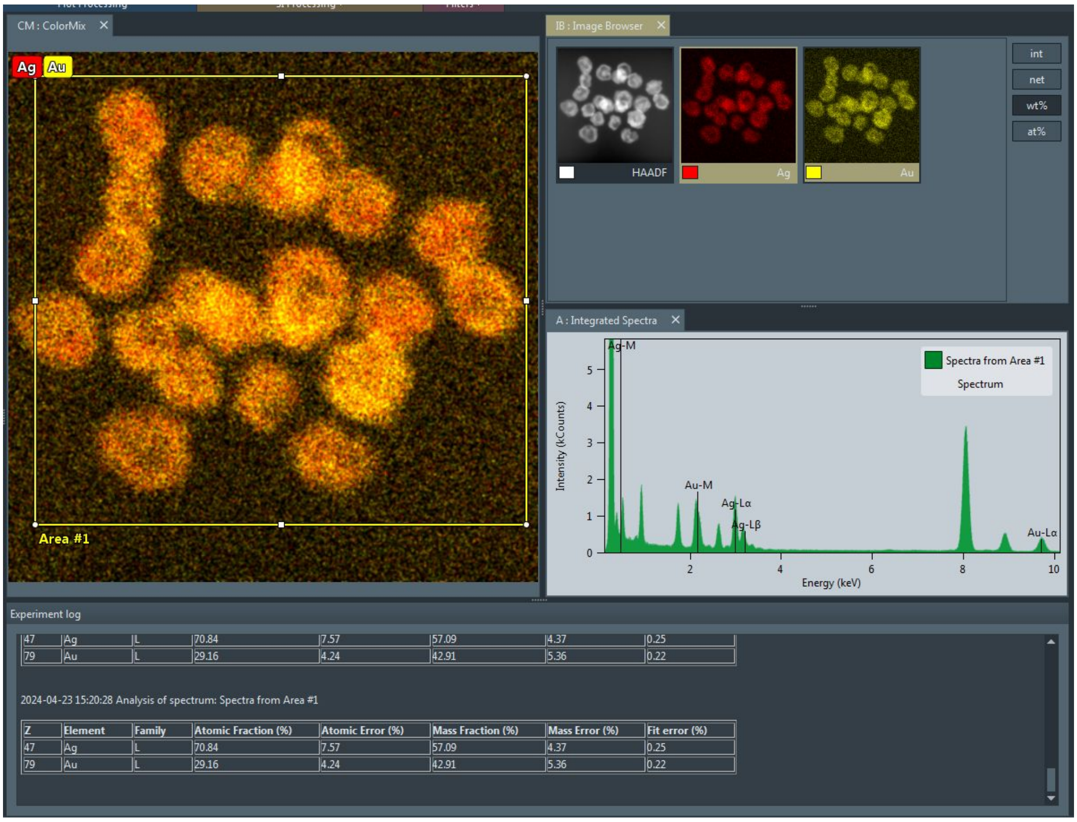

Figure S20. Elemental analysis and EDX spectrum of $Ag_{70}Au_{30}$NP CTAC sample.

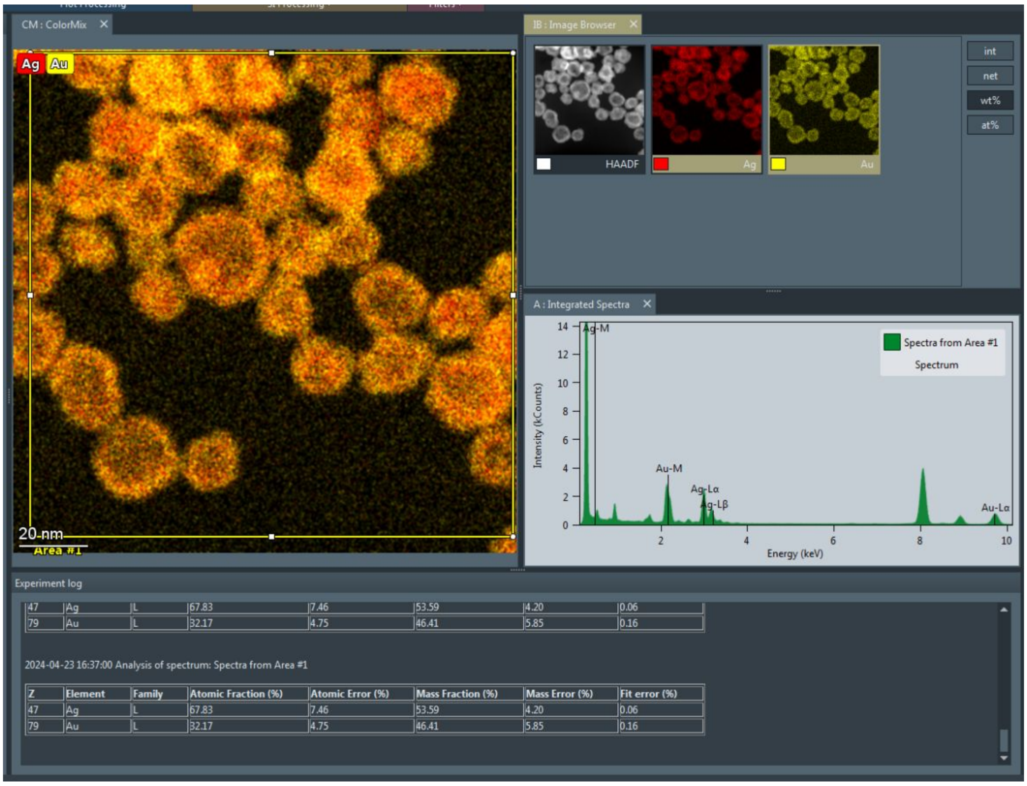

Figure S21. Elemental analysis and EDX spectrum of $Ag_{70}Au_{30}$NP NaCl sample.

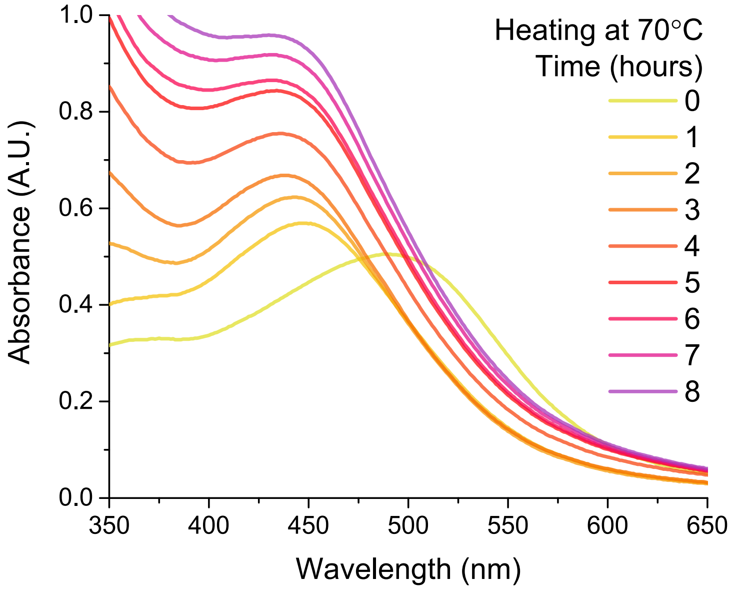

Figure S22. Absorbance spectra of the $Ag_{70}Au_{30}$NP CTAC sample heated at 70°C over time.

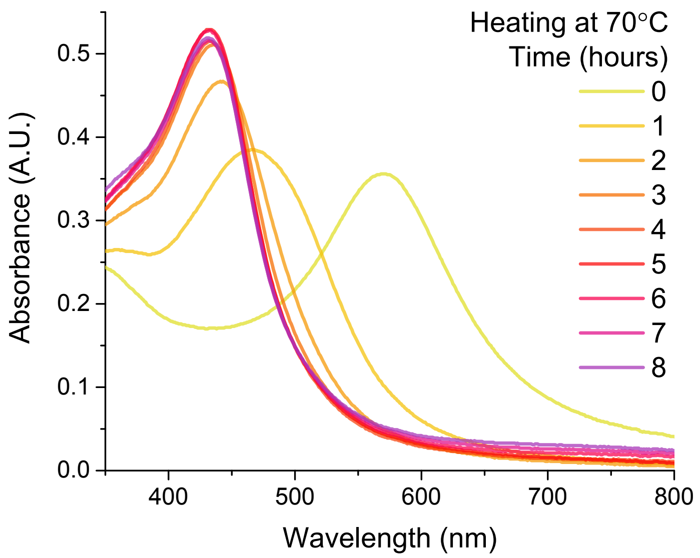

Figure S23. Absorbance spectra of the $Ag_{70}Au_{30}$NP NaCl sample heated at 70°C over time.

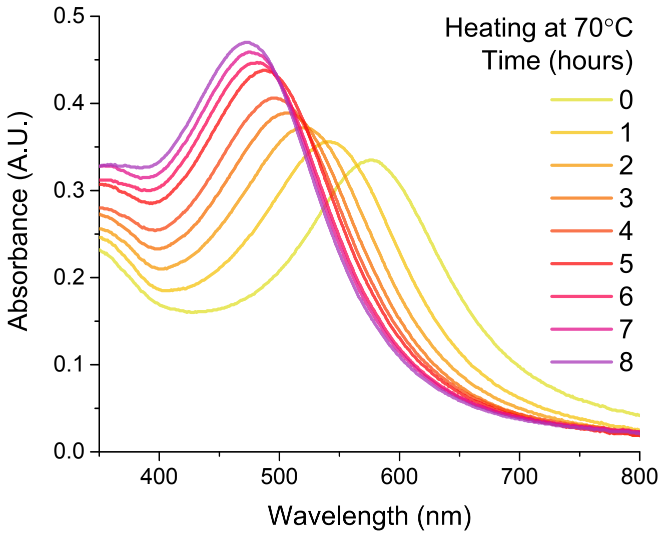

Figure S24. Absorbance spectra of the $Ag_{70}Au_{30}$NP NaCl + 10 μM GSH sample heated at 70°C over time.

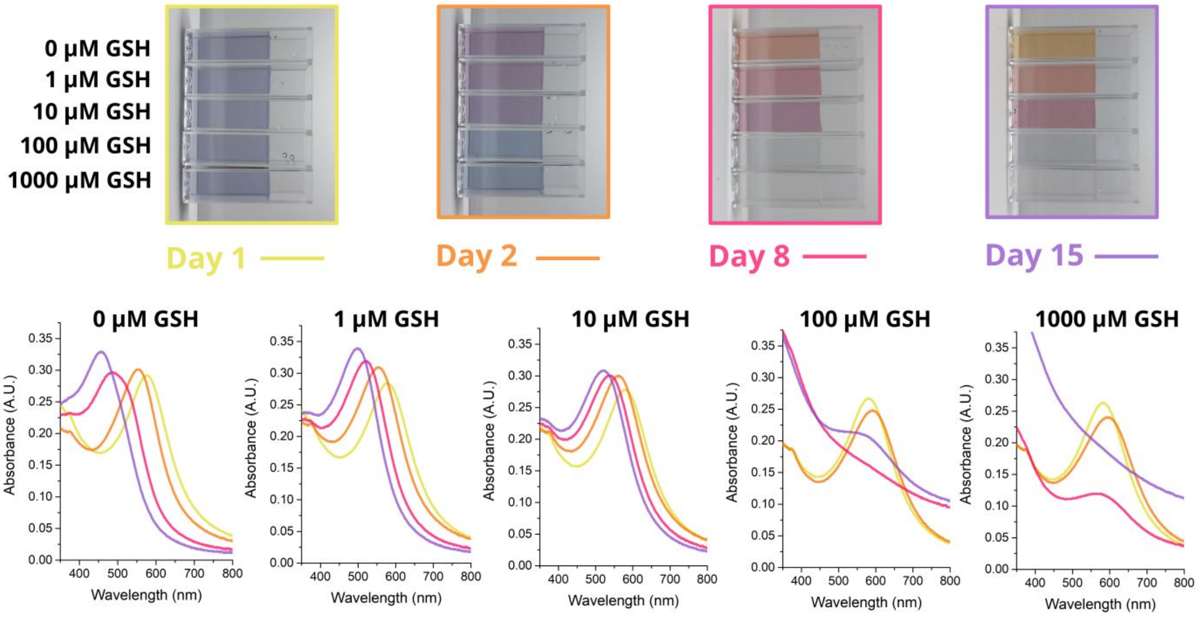

Figure S25. Photographs and absorbance spectra of $Ag_{70}Au_{30}NP$ mixed with different amounts of GSH and monitored over a period of 15 days while being stored at 25°C.

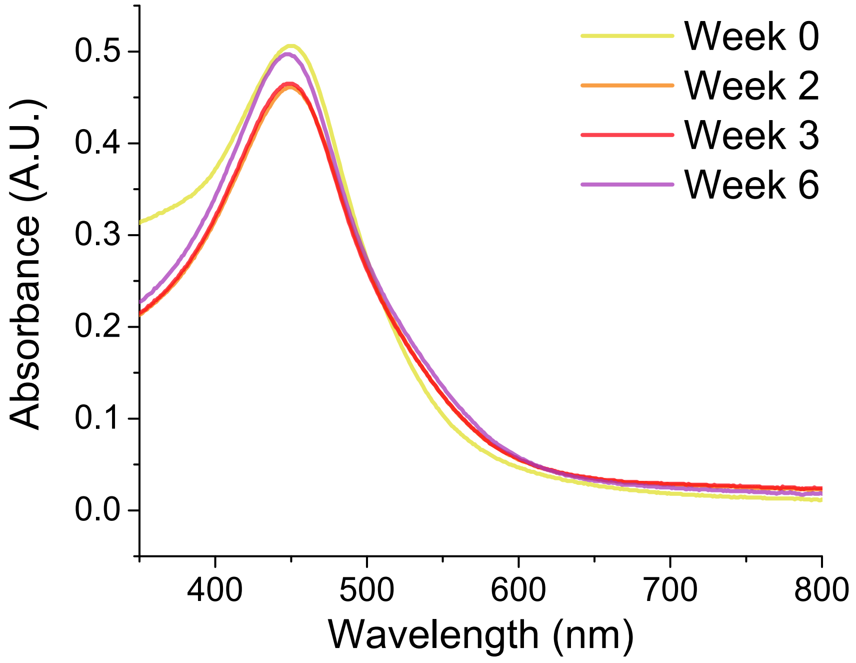

Figure S26. Absorbance spectra of heated $Ag_{70}Au_{30}NP$ NaCl sample over a period of 6 weeks while being stored at 25°C.

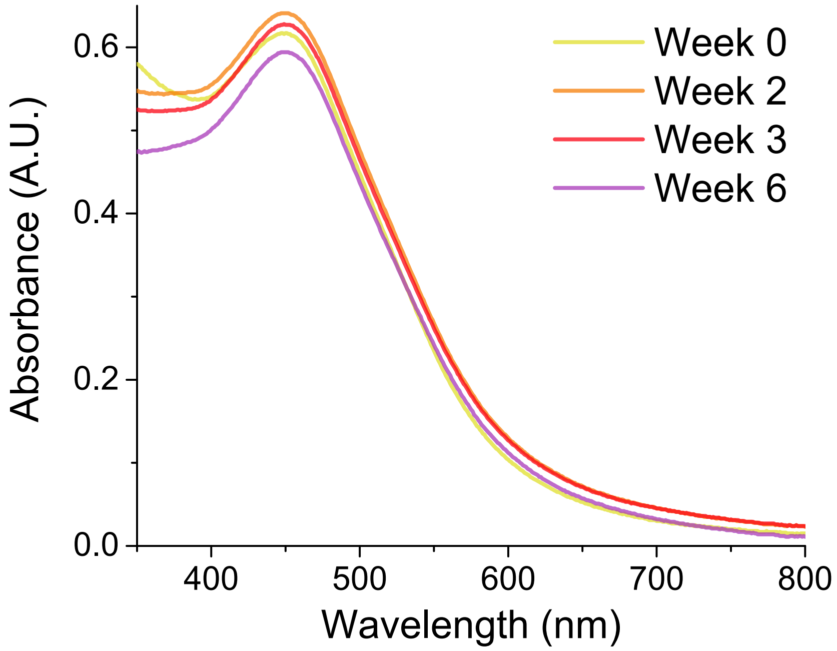

Figure S27. Absorbance spectra of heated $Ag_{70}Au_{30}$NP CTAC sample over a period of 6 weeks while being stored at 25°C.

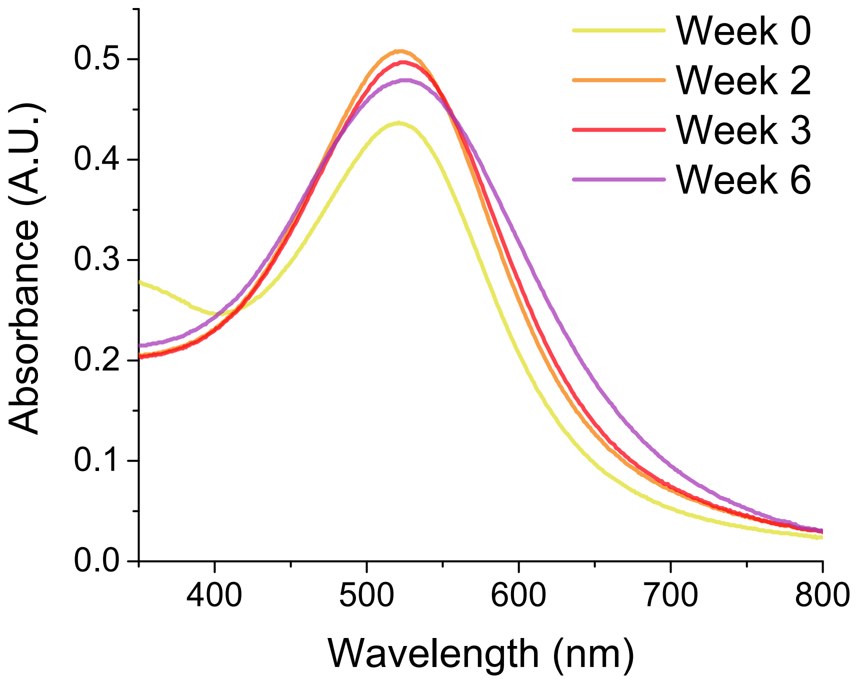

Figure S28. Absorbance spectra of heated $Ag_{70}Au_{30}$NP NaCl + 10 μM GSH sample over a period of 6 weeks while being stored at 25°C.

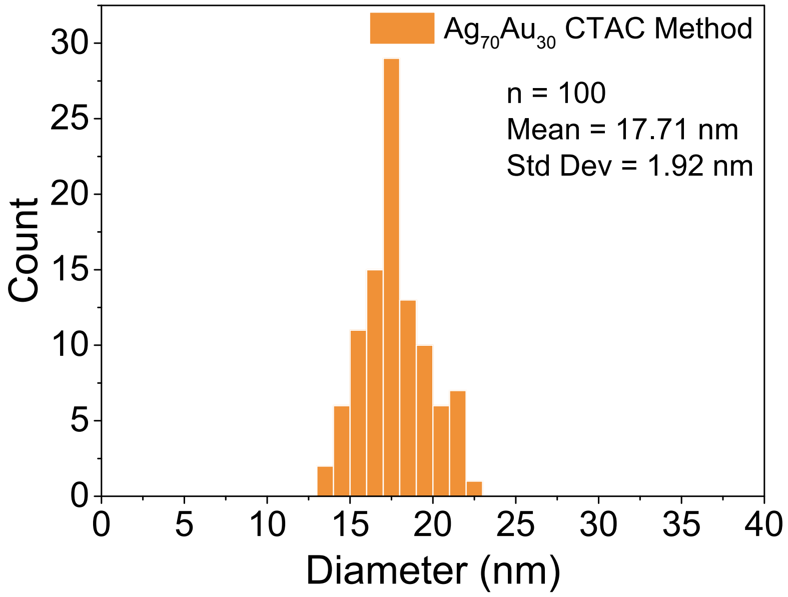

Figure S29. Size distribution of heated $Ag_{70}Au_{30}$NP CTAC sample.

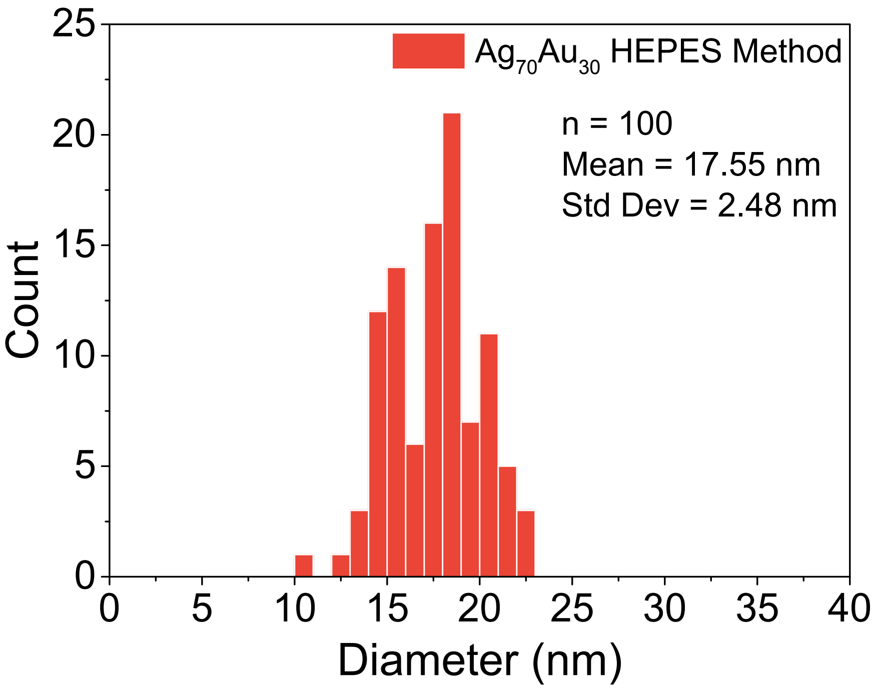

Figure S30. Size distribition of heated $Ag_{70}Au_{30}$NP NaCl sample.

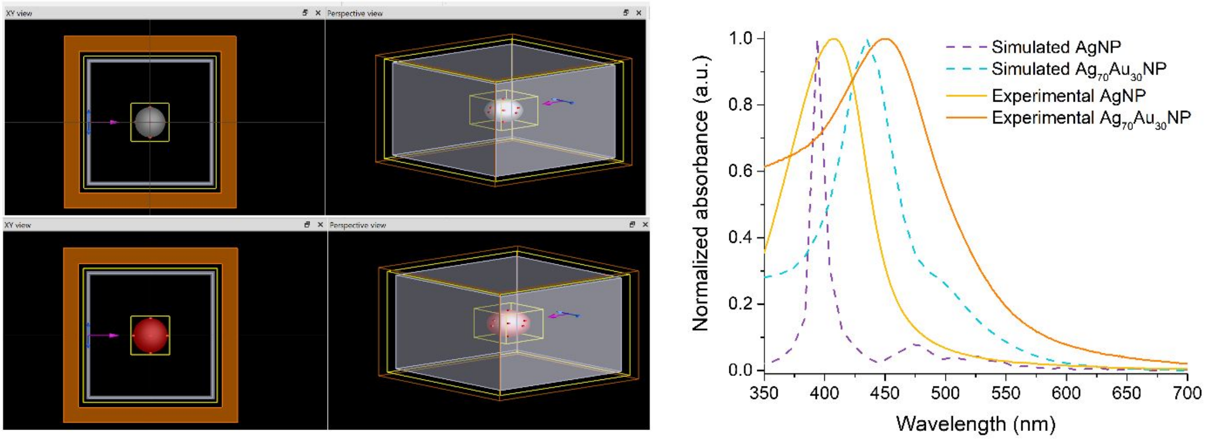

Figure S31. Lumerical FDTD models and simulated absorbance spectra for 16 nm AgNP and 18 nm $Ag_{70}Au_{30}NP$ in comparison with experimental results.

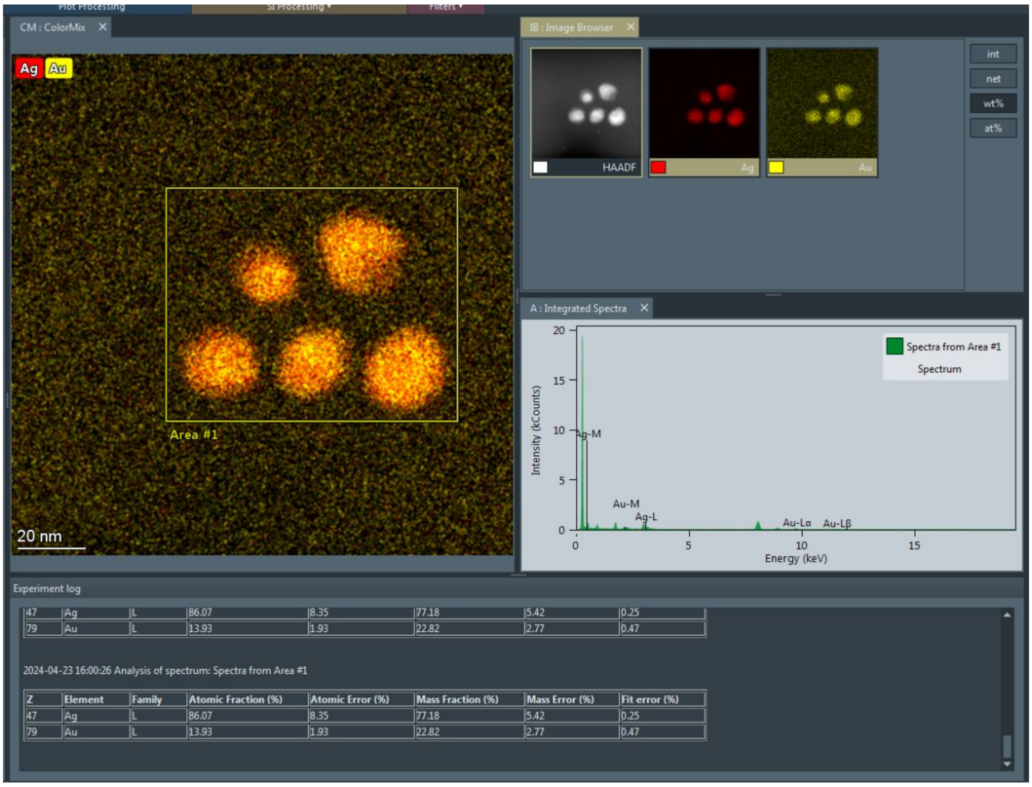

Figure S32. Elemental analysis and EDX spectrum of heated $Ag_{70}Au_{30}NP$ CTAC sample.

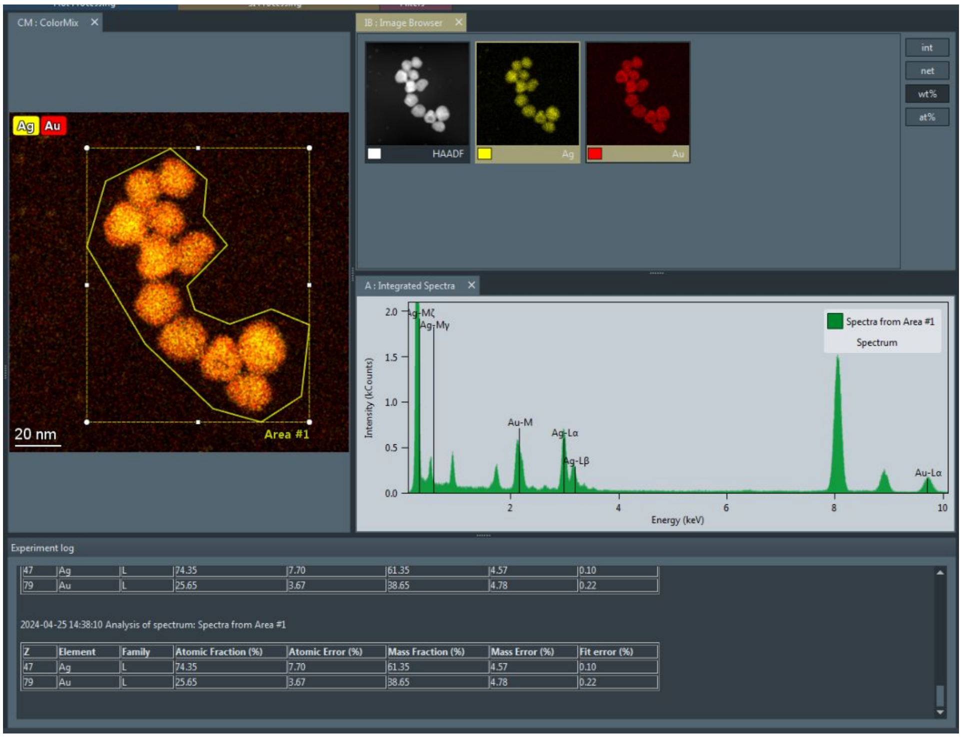

Figure S33. Elemental analysis and EDX spectrum of heated $Ag_{70}Au_{30}$NP NaCl sample.

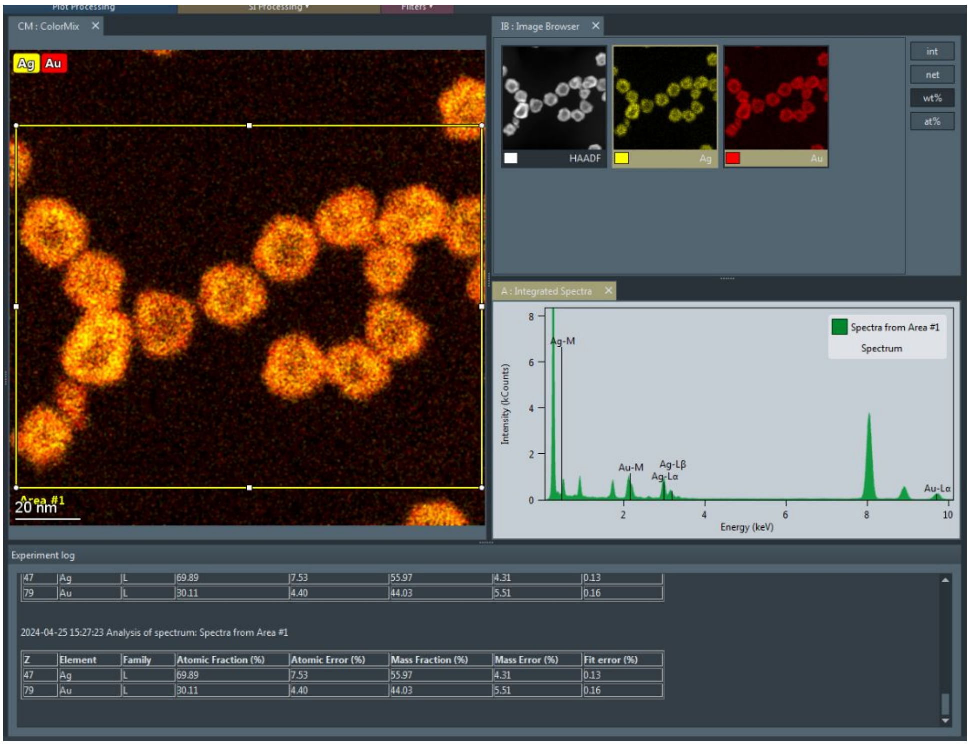

Figure S34. Elemental analysis and EDX spectrum of heated $Ag_{70}Au_{30}$NP NaCl + 10 μM sample.

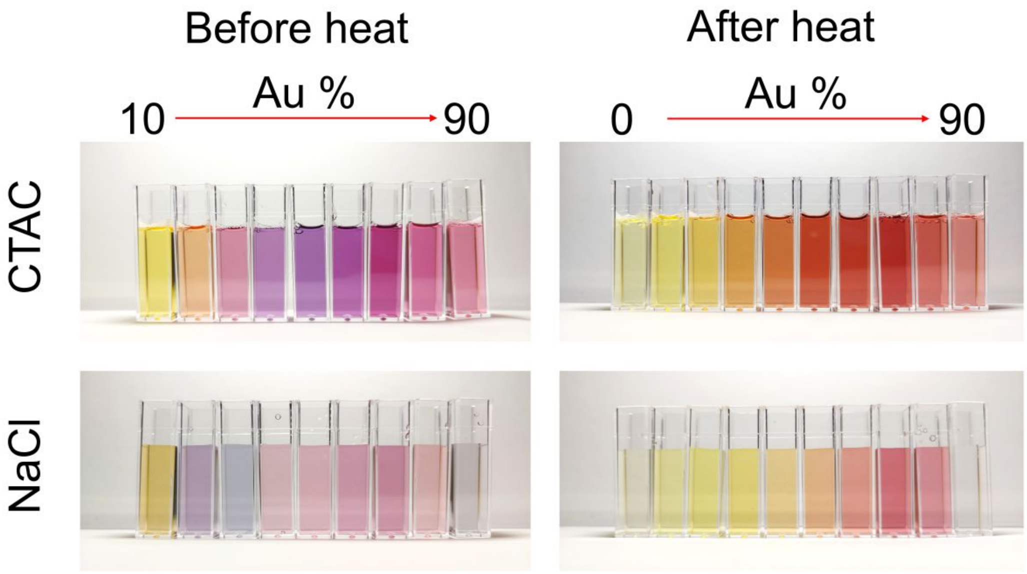

Figure S35. Photographs of AgAuNP made using CTAC and NaCl methods across a range of Au percentages, both before and after heating at 70°C overnight for 20 hours.

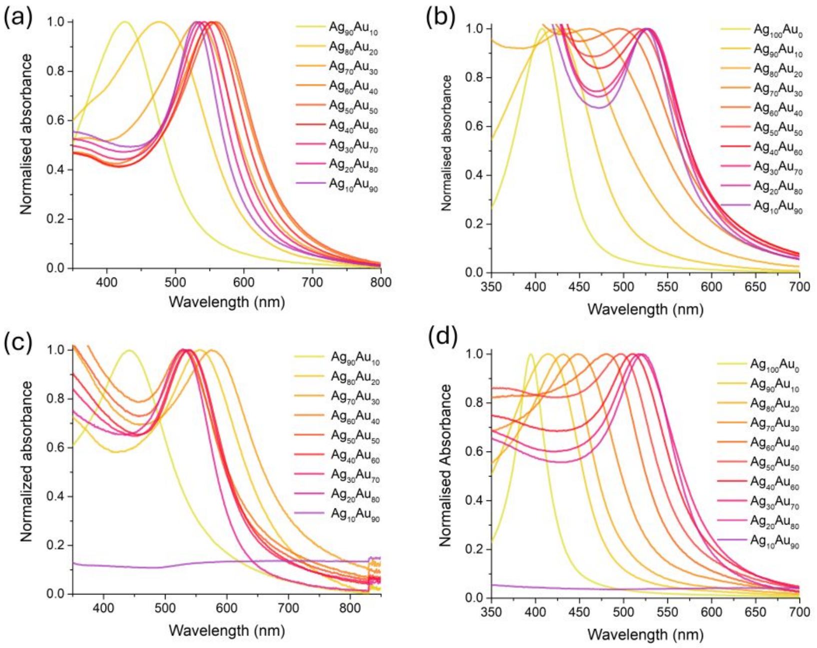

Figure S36. Normalised absorbance spectra of AgAuNP made using CTAC (a) before and (b) after heating at 70°C overnight for 20 hours. In addition to those made using the NaCl method (c) before and (d) after heating at 70°C overnight for 20 hours.

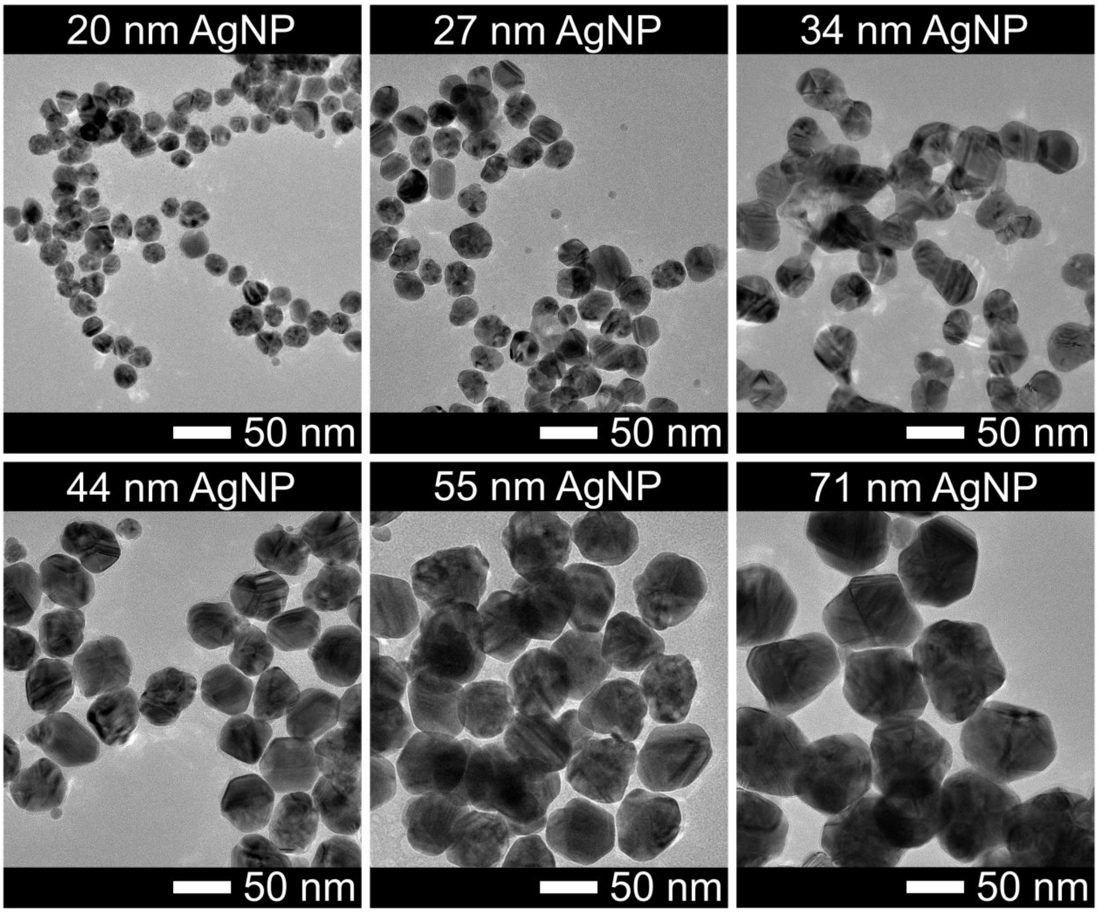

Figure S37. TEM images of AgNP prepared by sequential growth steps.

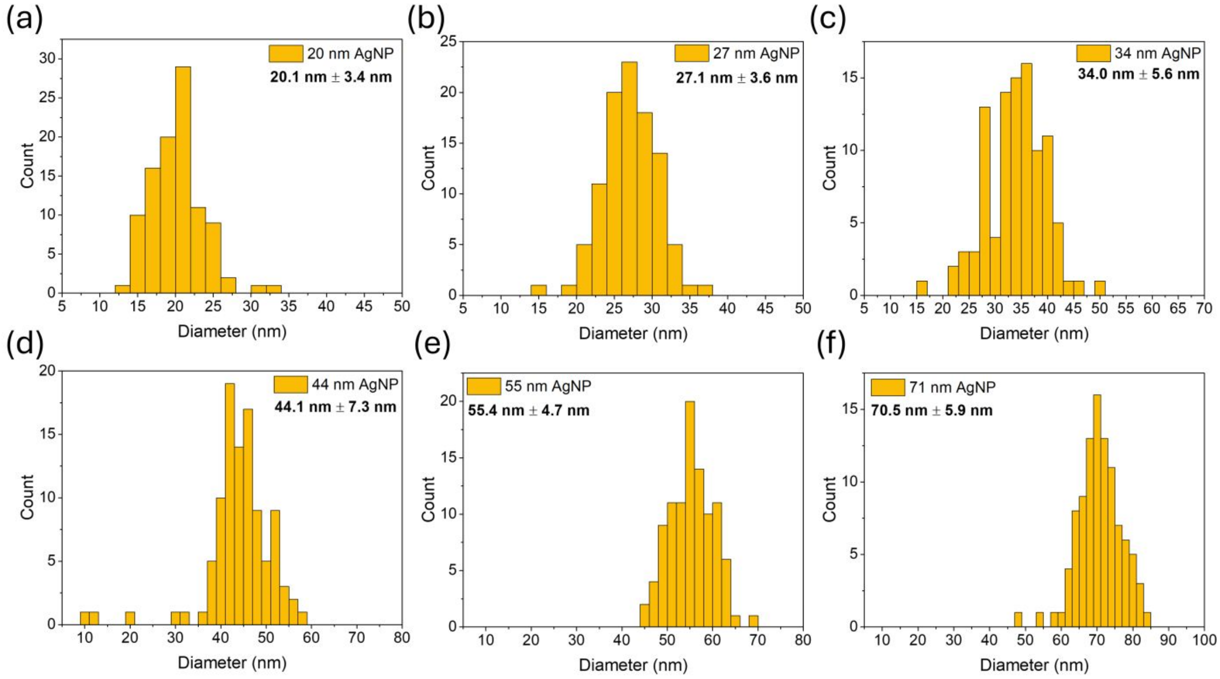

Figure S38. Size distribution of AgNP prepared by sequential growth steps.

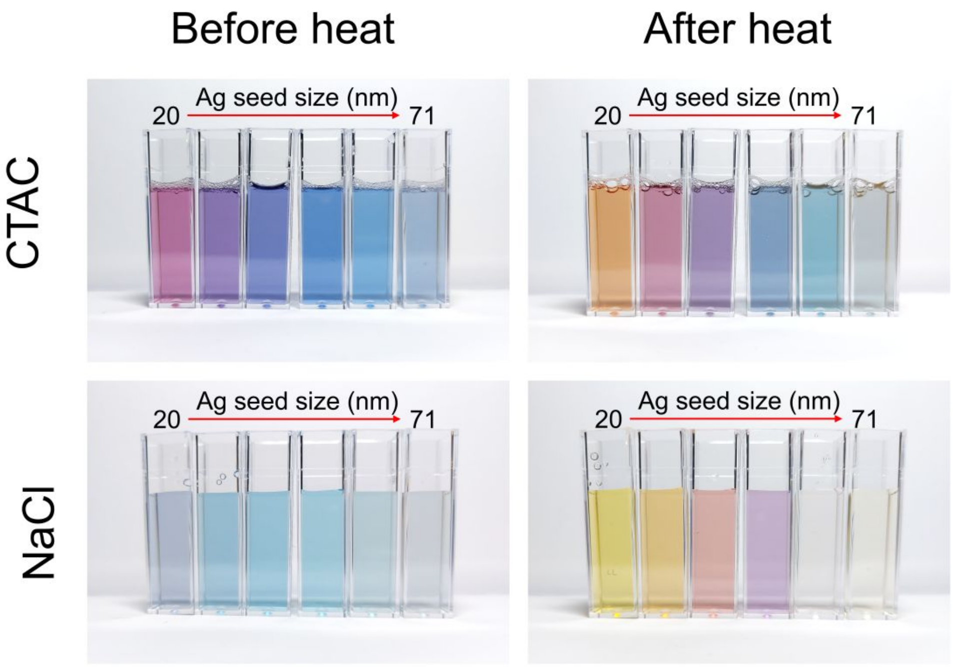

Figure S39. Photographs of $Ag_{70}Au_{30}NP$ made using CTAC and NaCl methods across a range of AgNP seed sizes, both before and after heating at 70°C overnight for 20 hours.

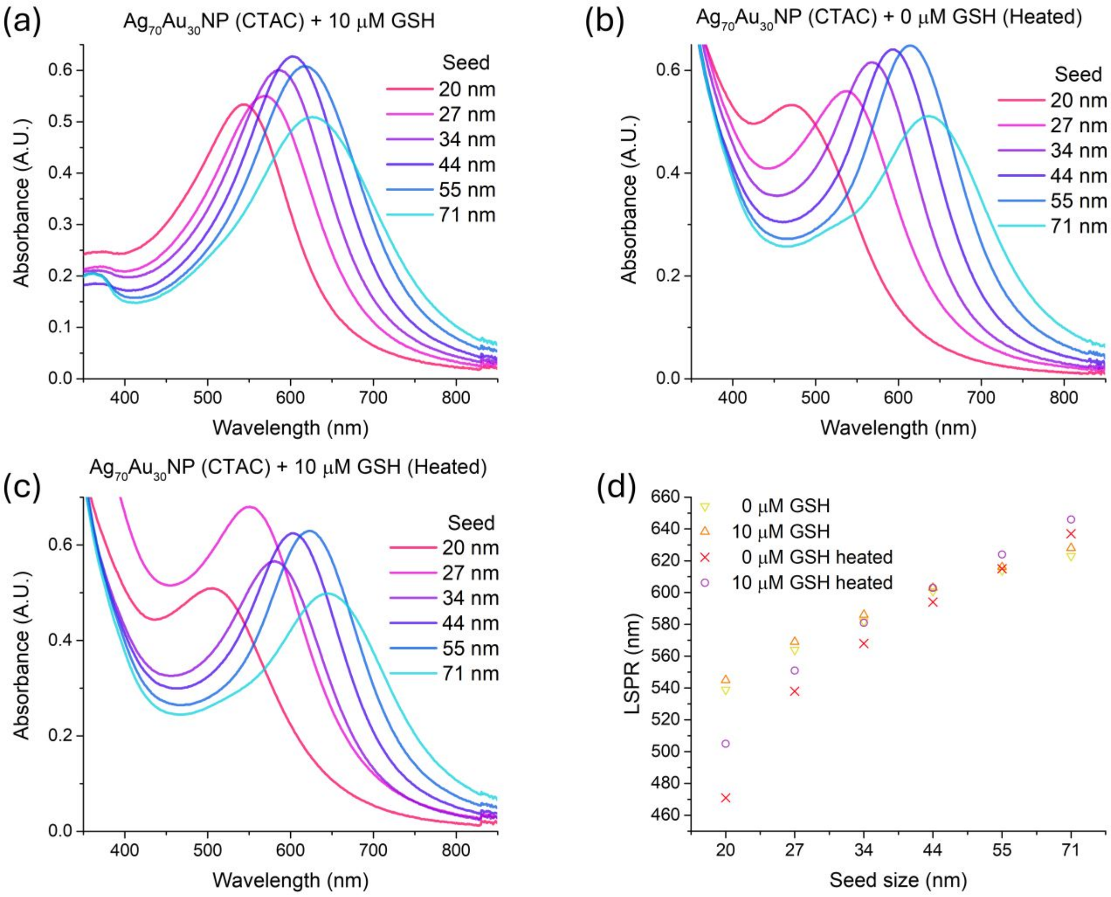

Figure S40. Absorbance spectra of $Ag_{70}Au_{30}NP$ made using different AgNP seed sizes. (a) CTAC + 10 μM GSH before heat, (b) CTAC + 0 μM GSH heated for 20 hours at 70°C and (c) CTAC + 10 μM heated for 20 hours at 70°C GSH. (d) Scatter plot of the peak LSPR for each $Ag_{70}Au_{30}NP$ sample across different seed sizes.

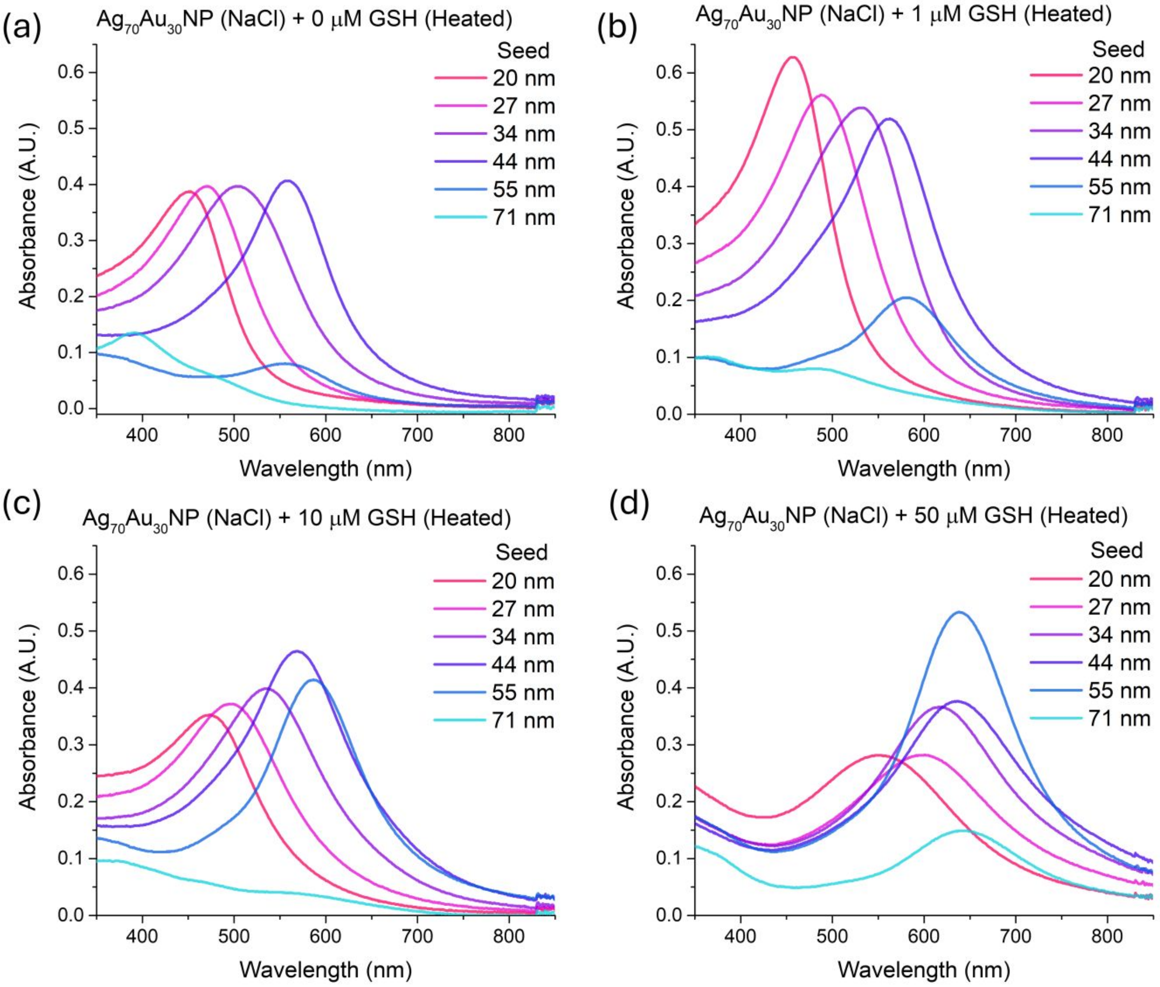

Figure S41. Absorbance spectra of $Ag_{70}Au_{30}NP$ made using the NaCl method with different AgNP seed sizes after heating for 20 hours at 70°C containing (a) 0 μM GSH, (b) 1 μM GSH, (c) 10 μM GSH and (d) 50 μM GSH.

References

(1) Polyanskiy, M. N. Refractiveindex.Info Database of Optical Constants. *Scientific Data 2024 11:1* 2024, *11* (1), 1–19. https://doi.org/10.1038/s41597-023-02898-2.

(2) Rioux, D.; Vallières, S.; Besner, S.; Muñoz, P.; Mazur, E.; Meunier, M.; Rioux, D.; Vallières, S.; Besner, S.; Meunier, M.; Muñoz, P.; Mazur, E. An Analytic Model for the Dielectric Function of Au, Ag, and Their Alloys. *Adv Opt Mater* 2014, *2* (2), 176–182. https://doi.org/10.1002/ADOM.201300457.
